# CpG Islands Undermethylation in Human Genomic Regions under Selective Pressure

**DOI:** 10.1371/journal.pone.0023156

**Published:** 2011-08-02

**Authors:** Sergio Cocozza, Most. Mauluda Akhtar, Gennaro Miele, Antonella Monticelli

**Affiliations:** 1 Gruppo Interdipartimentale di Bioinformatica e Biologia Computazionale, Università di Napoli “Federico II” - Università di Salerno, Naples, Italy; 2 Dipartimento di Biologia e Patologia Cellulare e Molecolare “L. Califano”, Università degli Studi di Napoli “Federico II”, Naples, Italy; 3 Dipartimento di Scienze Fisiche, Università degli Studi di Napoli “Federico II”, Naples, Italy; 4 Istituto Nazionale di Fisica Nucleare – Sezione di Napoli, Naples, Italy; 5 Istituto di Endocrinologia ed Oncologia Sperimentale, CNR Napoli, Naples, Italy; Ohio State University Medical Center, United States of America

## Abstract

DNA methylation at CpG islands (CGIs) is one of the most intensively studied epigenetic mechanisms. It is fundamental for cellular differentiation and control of transcriptional potential. DNA methylation is involved also in several processes that are central to evolutionary biology, including phenotypic plasticity and evolvability. In this study, we explored the relationship between CpG islands methylation and signatures of selective pressure in Homo Sapiens, using a computational biology approach. By analyzing methylation data of 25 cell lines from the Encyclopedia of DNA Elements (ENCODE) Consortium, we compared the DNA methylation of CpG islands in genomic regions under selective pressure with the methylation of CpG islands in the remaining part of the genome. To define genomic regions under selective pressure, we used three different methods, each oriented to provide distinct information about selective events. Independently of the method and of the cell type used, we found evidences of undermethylation of CGIs in human genomic regions under selective pressure. Additionally, by analyzing SNP frequency in CpG islands, we demonstrated that CpG islands in regions under selective pressure show lower genetic variation. Our findings suggest that the CpG islands in regions under selective pressure seem to be somehow more “protected” from methylation when compared with other regions of the genome.

## Introduction

DNA methylation at CpG sites is one of the most intensively studied epigenetic mechanisms [1] . CpG sites are DNA regions where a cytosine nucleotide occurs next to a guanine nucleotide. Cytosines in CpG dinucleotides can be methylated to form 5-methylcytosine. Human genome contains about 30 million CpGs that exist in a methylated or unmethylated state. A part of all CpG sites present in the genome are clustered into CpG islands that are defined as genomic regions with increased CpG density. These CGIs are enriched at genes, about 60% of all genes in the human genome containing a CpG island upstream [2]. The methylation status of CGIs can influence gene expression [3] [1]. The hypermethylation at promoter CGIs typically results in a decreased transcription of downstream genes [4]. Further, aberrant DNA methylation has been often reported to cause various human diseases [5] [6] [7].

Three DNA methyltransferases, namely DNMT1, DNMT3a, and DNMT3b [8] are involved in the maintenance of DNA methylation during the cell cycle. When the two parental DNA strands are separated in the S-phase of the mitosis, two hemimethylated strands are produced. DNMT1 is a component of a protein complex with high affinity with hemimethylated DNA, subsequently restoring methylation on the daughter strands [9]. Also demethylation is an important biological mechanism, as illustrated, for example, by the demethylation of the paternal and maternal genomes in the zygote after fertilization [10] or by the reprogramming of pluripotency cells to differentiated cells [11]. Nevertheless, the molecular mechanism of DNA demethylation in mammals is disputed, one possibility being that cells demethylate their genome by passive demethylation.

Several evidences suggest a dependence of DNA methylation on local sequence content [12]. DNA methyltransferases within eukaryotic cells are not free, but they are compartmentalized by interaction with nuclear components [13]. Thus it is likely that chromatin structure of a genomic region will have an important impact on the maintenance of methylation of that region. It could be hypothesized that there are genomic regions somehow “protected” in vivo from methylation but yet readily accessible to exogenously added soluble DNA methylases [14].

Nonetheless, a complete understanding of the role of DNA methylation and the mechanisms responsible for its establishment and maintenance remain elusive [1].

Many studies focused on the interplay between epigenomic regulation and evolution, because DNA methylation is involved in several processes that are central to evolutionary biology, including phenotypic plasticity and evolvability [15]. Changes in the regulation of gene expression levels have long been hypothesized to play an important role in evolution [16]. Nevertheless, studies specifically addressed to the relation between promoter methylation and selective pressure in Homo Sapiens are still lacking.

Several tools are needed to study the relation between CGIs methylation and selective pressure in a genomic perspective. First, we need tools that recognize genomic signals of selective pressure. Many methods have been developed to exploit signatures left by natural selection, each signature providing distinct information about selective events [17]. Since one of the main effects of selection is to modify the levels of variability within and between species, these methods could be roughly classified into two groups. To the first group belong the methods that use a population genetic approach, while to the second group belong methods that use a comparative approach. While population genetic approaches aim to detect recent selection events occurring in a population, comparative approaches, involving data from multiple different species, are suitable for detecting more ancient selections [17]. By these methods, hundreds of such regions putatively under selective pressure have been identified. They are typically as large as few hundreds of kilobases to megabases, and may contain many genes.

The second requirement to study the relation between CGIs methylation and evolution is the availability of methylation data at genomic scale. Recent advances in high-throughput sequencing technologies are enabling epigenetics to progress rapidly into an ‘omic’ science [18]. In particular, the Encyclopedia of DNA Elements (ENCODE) Consortium [19], [20] is providing masses of methylation data that may be accessed and used by the entire scientific community. The analysis of these relevant datasets by computational methods could complement experimental approaches to further our understanding of DNA methylation [21] [22].

In this study, we explored the relationship between CGIs methylation and signatures of selective pressure in Homo Sapiens, using a computational methodology.

We compared the CGIs methylation level in genomic regions under selective pressure with CGIs localized in the remaining genome. We evaluated DNA methylation levels both by direct analysis of CpG methylation in cell lines and by an indirect approach that uses the analysis of genetic variation inside CGIs.

To define genomic regions under selective pressure, we used three different methods oriented to provide information about selective events happened in different periods of human evolution.

Independently of the methods used both to evaluate CGIs methylation and to estimate selective pressure, we found evidences of undermethylation of CGIs in human genomic regions that undergone selection.

## Results

### DNA methylation in cell lines and signatures of selective pressure

Based on datasets available in public repository we estimated the CGIs methylation in 25 cell lines.

Genomic coordinates of 28,691 CGIs were obtained from UCSC Genome Browser “CpG Islands” track. As known, USCS CGIs file contains also data related to sequence for alternative haplotypes (present mainly in chr6, for the inclusion of alternative versions of the MHC region). Of course, in our analysis we filtered the file excluding these duplicated data. Excluding CGIs corresponding to sequences for alternative haplotypes, we obtained 27.718 unique CGIs. Cell line methylation data were obtained by downloading them from UCSC Genome Browser “HAIB Methyl RRBS” track. This track reports the percentage of DNA molecules that show cytosine methylation at specific CpG dinucleotides in several cell lines. The 25 cell lines that we used could be roughly divided in three groups: cancer transformed cells (n = 6), EBV transformed cells (n = 2) and normal untransformed cells (n = 17). The complete list of the cell used, with their characteristics are shown in Table S1. We extracted only the methylation values of those CpGs that were localized inside CGIs (order 10^5^ per cell line).

To estimate the methylation of each CpG island we calculated the mean of all CpGs methylation values into a CpG island. We were able to estimate the methylation status of about 10^4^ CGIs for each cell line. Table S2 lists, for each cell type, the description of the CpGs analyzed. As expected, the CGIs mean methylation values were higher in Cancer Transformed (mean = 26.91, SE = 2.84) and lower in Normal Untransformed cells (mean = 14.34, SE = 0.57), EBV transformed cell showing intermediate levels (mean = 18.93, SE = 1.46) (Figure S1).

To explore the possible relationship between CGIs methylation and selective pressure we compared the methylation of the CGIs inside genomic regions showing signature of selective pressure with the methylation of the CGIs in the remaining genomic regions.

To obtain genomic regions with signatures of selective pressure, we used three different approaches.

As first approach, we used the per-continent Integrated Haplotype Score (iHS) [23]. This score belongs to the Extended Haplotype Homozygosity (EHH) statistic “family”, proposed by Sabeti et al. [24]. The iHS measures the decay of identity, as a function of distance, of haplotypes that carry a specified “core” allele at one end and it is considered a measure of recent positive selection. The normalized iHS scores (see materials and methods) were obtained from UCSC Genome Browser “HGDP iHS” track.

To define genomic regions putatively under selective pressure by this method, we scanned normalized iHS scores across the whole genome and selected the genomic intervals where iHS score values ≥2. Once detected such compact regions, we extended their boundaries to the nearest loci where iHS was exactly vanishing. According to these criteria, 586 regions were identifies. We denoted these regions as “High iHS regions” (HIR). Table S3 reports the HIRs that we identified and their boundaries.

Next we identified CGIs localized within HIRs. We found that 2,545 CGIs were localized inside HIRs whereas the remaining 26,146 were placed outside. We compared the methylation of CGIs inside HIRs with the methylation of CGIs localized outside these regions.

**Figure 1:**
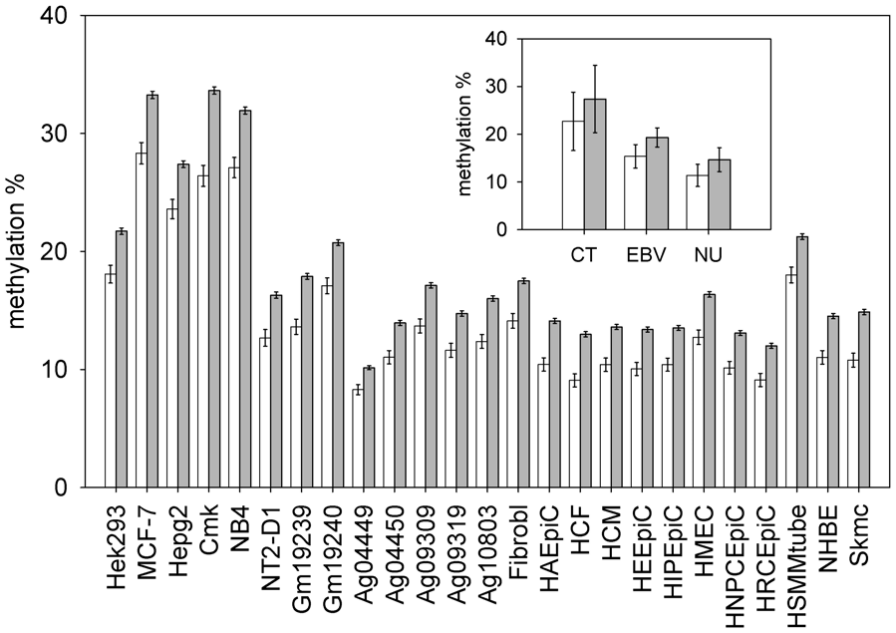
Methylation of HIR CGIs compared to methylation of CGIs in other genomic regions. For each cell line, the mean methylation value of CGIs inside HIR regions (open bars) and of the CGIs in the remaining part of the genome (closed bars) are reported. Inset shows the same data summarized by cell group (Cancer Transformed = CT, EBV transformed = EBV, Normal Untransformed = NU). Values are means +/− Standard Error (SE).

Figure 1 shows the results obtained. In all cell lines analyzed, the CGIs inside HIR regions were less methylated than the CGIs in the remaining part of the genome. The differences were highly statistical significant (Bootstrap p-value≤10^−4^) in all cell lines analyzed. Table S4 reports in detail the results of this analysis. The Bootstrap procedure adopted to evaluate the difference between means of distributions is described in Materials and Methods.

An additional method able to detect regions putatively under selective pressure is represented by the Selective Sweep Scan (S) score, which is based on the comparison of Homo Sapiens DNA with Neanderthal DNA [25]. This score, when positive, indicates more derived alleles in Neanderthal than expected, given the frequency of derived alleles in human. On contrary, a negative score indicates fewer derived alleles in Neanderthal, and may suggest an episode of positive selection in early humans, after divergence with Neanderthal and before human populations divergence. We used the 212 regions with S scores in the lowest 5% of the distribution (5% Lowest S Regions, 5LSR) contained in the UCSC Genome Browser (see materials and methods). Table S5 reports the regions used with their relative boundaries.

**Figure 2:**
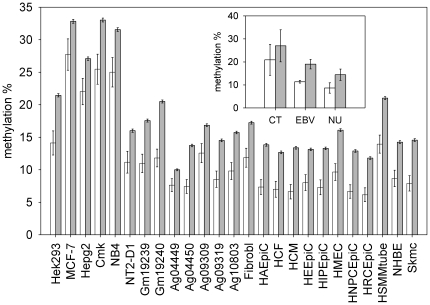
Methylation of 5LSRs CGIs compared to methylation of CGIs in other genomic regions. For each cell line, the mean methylation value of CGIs inside 5LSRs regions (open bars) and of the CGIs in the remaining part of the genome (closed bars) are reported. Inset shows the same data summarized by cell group (Cancer Transformed = CT, EBV transformed = EBV, Normal Untransformed = NU). Values are means +/− SE.

We found that 348 CGIs were localized inside 5LSRs and the remaining 28,343 outside them. Figure 2 shows the results obtained by comparing the methylation of CGIs inside 5LSRs with the methylation of CGIs localized in the other regions of the genome.

Also for this different measure of selective pressure, in all cell lines analyzed, CGIs inside regions under selective pressure were less methylated than the remaining CGIs. The differences were highly statistical significant (Bootstrap p-value<10^−3^) in 17 cell lines analyzed, but did not reach this significance in 8 cell lines (p<0.05). Nevertheless, combining the results of all 25 cell lines by means of the test statistic - 2 log (p_1_,p_2_ … p_25_), where p_1_, p_2_ … p_25_ are the p-values of the individual tests, we reached a combined statistical significance much less than 10^−3^. Table S6 reports in detail the results of the analysis

To check if the results could be due to the same CGIs identified by both methods, we searched for CGIs that are both within HIRs and within 5LSRs. We found only 70 CGIs in common between these two groups, indicating that the results obtained by the two methods are driven by different sets of CGIs. In addition, excluding these 70 CGIs from the analysis, the result continued to be highly significant both for HIRs and 5LSRs (data not shown). It is intriguing to note that these 70 CGIs were less methylated when compared both to the remaining HIR CGIs and 5SLR CGIs, but the differences were not statistical significant (data not shown).

To further define regions under selective pressure, we decided to use a third and last approach that looks for sequences that are conserved across species [26]. By this approach, conserved regions are defined as genomic regions with a reduced rate of evolution compared to what is expected under neutral drift. Several methods for detecting conserved regions in multiple alignments have been described. We used data downloaded from UCSC Genome Browser Conservation (cons46way) Track, which lists 725,627 Conserved Elements (CEs) that were predicted to be conserved among primates [27].

We Identified 26,936 CEs located inside 14,391 CGIs, by filtering all genomic CEs by CGIs. Excluding CGIs corresponding to sequences for alternative haplotypes, we obtained 13.288 unique CGIs containing 25.362 CEs. We named “CE CpG islands (CE CGIs)” those CGIs that contain at least one conserved element. For each cell line, we compared the methylation of CE CGIs with the methylation level of the remaining CGIs not containing conserved elements.

**Figure 3:**
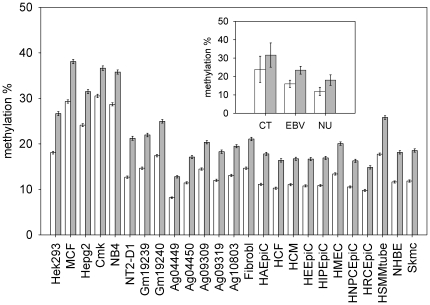
Methylation of CE CGIs compared to methylation of CGIs that do not contain conserved elements. For each cell line, the mean methylation value of CE CGIs (open bars) and of the CGIs that do not contain conserved elements (closed bars) are reported. Inset shows the same data summarized by cell group (Cancer Transformed = CT, EBV transformed = EBV, Normal Untransformed = NU). Values are means +/− SE.

In all the cell lines analyzed, CE CGIs were less methylated than CGIs that do not contain conserved elements (Figure 3). The differences were highly statistical significant (Bootstrap p-value<10^−4^) in all cell lines analyzed. Table S7 reports in detail the results of this analysis.

Since the number of CE CGIs is higher than that of HIR CGIS and 5SLR CGIs, it could be possible that all HIR CGIs and 5SLR CGIs are contained in the CE CGI group. In this case the results we found with HIR and 5SLR could be due to CE only.

**Figure 4:**
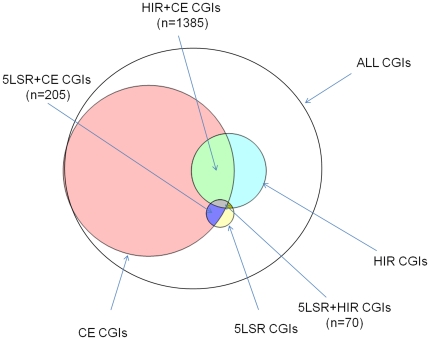
Venn diagram showing the overlaps among CGIs localized in the regions under selective pressure detected by the three methods used.

To check this possibility, we estimated the overlaps between the CGIs lists obtained by the different methods (Figure 4). We found that 1,385 CGIs were in common between CE and HIR (HIR+CE CGIs) and 205 were in common between CE and 5SLR (5SLR+CE CGIs). If the phenomena underlying the three signatures (CE, HIR and 5SLR) contributed independently to lower the CGIs methylation, we expected that CGIs in regions with two signatures of selective pressure showed lower methylation when compared to CGIs in regions with one signature only. We found that, in all cell lines analyzed, HIR+CE CGIs were less methylated than the remaining CE CGIs. The differences were highly statistical significant (Bootstrap p-value<10^−3^) in 14 cell lines analyzed, but did not reach this significance in 11. In these eleven cell lines the differences were significant only at p<0.05 (Figure S2, Table S8). Also 5SLR+CE CGIs were less methylated when compared to the remaining CE CGIs, in all cell lines analyzed. The differences were highly statistical significant (Bootstrap p-value<10^−3^) in 17 cell lines, but did not reach this significance in 8. In these eight cell lines the differences were significant only at p<0.05 (Figure S3, Table S9). Also in these two cases the joint analysis of all cell lines yielded a combined statistical significance much less than 10^−3^.

In the genome, CGIs are located in 5′,3′ or in other gene regions, as well as in intergenic regions. We decided to estimate the methylation of CGIs located in these different locations to assess if the CGIs undermethylation that we found in regions under selective pressure is restricted to CGIs with a specific localization. We used the 4 classes of CpG islands described by Medvedeva et al. [28] : 5′ CGIs (in 5′-flank region, 5′ UTR-exon , 5′UTR-intron , initial coding exon and initial intron), intragenic CGIs (in internal exons and internal introns), 3′ CGIs (in final exons, final introns, 3′ UTR exons and 3′ UTR introns) and intergenic CGIs (located at least 3 kb from any known gene upstream and downstream). In particular, 5′ CGIs are located in regions that, starting 3 kb upstream Transcription Start Site, extend till the first intron. Considering all cell lines, 5′ CGIs showed the lowest methylation level (weighted mean = 9.01) , intragenic and 3′ CGIs showed the highest values (respectively, weighted mean = 55.21 and 42.59) and intergenic CGIs showed intermediate methylation values (weighted mean = 21.31). For each cell line, the differences among CGIs methylation of different genomic regions were high statistical significant (Kruskal-Wallis Test, p-value≤2.2 10^−16^) (Table S10).

Next we divided CGIs with signature of selective pressure according the above described classes. Unfortunately, for intragenic and 3′ classes, we did not obtain a number of HIR CGIs and 5LSR CGIs sufficient to perform a consistent statistical analysis. In particular, in these classes we found about 80 HIR CGIs and less than 10 5LSR CGIs.

We were able to perform statistical analysis only by using CE as signature of selective pressure. In all cell lines but 2 (which were both cancer cell lines), 5′ CGIs in CE regions were undermethylated when compared to 5′ CGIs located outside CE regions (Bootstrap p-value<10^−4^). Intragenic and 3′ CGIs located in CE regions showed no differences in methylation when compared to intragenic and 3′ CGIs outside CE regions. In all cell lines, intergenic CGIs in CE regions were severely undermethylated when compared to intergenic CGIs located outside CE regions (Bootstrap p-value p<10^−4^) (Table S11).

This first set of experiments suggested that, in different cell lines, the GCIs localized in genomic regions under selective pressure are undermethylated. CGIs in regions with two signatures of selective pressure (in which CE is involved) showed lower methylation when compared to CGIs in regions with one signature only. Furthermore, at least for CE, the CGIs undermethylation that we found in genomic regions under selective pressure is specifically due by CGIs located at the 5′ and in the intergenic regions.

### Genetic variation inside CpG islands and signatures of selective pressure

We decided to estimate the CGIs methylation by a different, indirect approach. It is well settled that 5-methylcytosine is the initial molecule in the deamination reaction that generates thymine; thus, methylation may be required for increased mutation rates at CpG sequences. We predicted that CGIs localized in regions under selective pressure, being less methylated, would be less likely to mutate. Under this hypothesis, these CGIs should show a lower degree of genetic variation among individuals.

To evaluate the degree of genetic variation in CGIs, we calculated the frequency of SNPs present in each CGI. Among the 26,033,053 SNPs from dbSNP (build 131), we selected the 199,514 SNPs that were located inside CGIs. To obtain a normalized value of SNP frequency for each CGI, we divided the number of SNPs present in each CGI by its size. By this method we were able to calculate the SNP frequencies for 25,558 CGIs.

We found that, on average, each CGI contained 1.04 SNP/100 bp (range 0.04–63.28).

Then we compared the SNP frequency of CGIs inside the regions under selective pressure with the SNP frequency of CGIs localized in the other regions of the genome.

**Figure 5:**
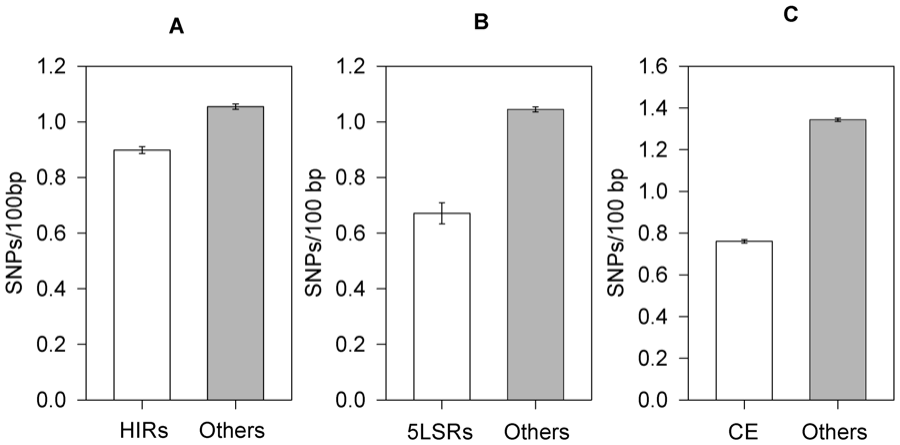
SNP content of CGIs in genomic regions under selective pressure compared with CGIs localized in other genomic regions. The mean SNP frequencies (SNPs/100 bp) of CGIs in genomic regions under selective pressure (open bars) and of CGIs localized in other genomic regions (closed bars) are reported. The regions are: A = HIR, B = 5SLR and C = CE). Values are means +/− SE.

Figure 5 reports the results obtained. The 2,345 CGIs localized in HIRs showed a mean of 0.89 SNP/100 bp in comparison with 1.05 of the other 23,213 CGIs (Bootstrap p-value<10^−4^). The 309 CGIs localized in 5LSRs showed a mean of 0.67 SNP/100 bp in comparison with 1.04 of the other 25,249 CGIs (Bootstrap p-value<10^−4^). The 13,286 CE CGIs showed a mean of 0.76 SNP/100 bp in comparison with 1.34 of the other 12,272 CGIs (Bootstrap p-value<10^−4^).

Also for this approach we checked if CGIs in regions with two signatures of selective pressure (HIR+CE or 5SLS+CE) showed differences compared to CGIs in regions showing only a signature (CE). We found that the 205 5SLR+CE CGIs contained less SNPs than the remaining 13,081 CE CGIs (mean = 0.61 SNP/100 bp vs. 0.76 SNP/100 bp, Bootstrap p-value<10^−4^). On contrary, the 1,386 HIR-CE CGIs did not show any difference in SNP content in comparison with the remaining 11,900 CE CGIs (mean = 0.74 vs. 0.76, Bootstrap p-value = 0.36).

In summary, we demonstrated that the CGIs localized in regions showing signatures of selective pressures contain less SNPs than CGIs in other regions of the genome. When compared to CGIs in regions with one signature only, CGIs in regions with two signatures of selective pressure showed differences in the case of 5SLR but not for HIR.

## Discussion

One of the most studied epigenetic modifications is the DNA methylation, which is evolutionarily ancient and associated with regulation of gene transcription [29]. DNA methylation could be central both to the ability of a population of organisms to change its phenotype in response to changes in the environment and to its ability to generate genetic diversity and evolve through natural selection [15]. The evolutionary conservation and divergence of epigenetic mechanisms in eukaryotes have started to be revealed by genetic and genomic studies of various organisms [29]. A general scenario that seems to emerge is that the epigenetic marks and the mechanisms that establish these marks are basically ancient and conserved, but the precise details of how these marks function within genomes is far to be completely clarified. An intriguing question is how evolutionary forces have adapted epigenetic mechanisms to the needs of the specific organism and, within a species, to the needs of a specific population.

In this study we searched for possible differences in DNA methylation between genomic regions under selective pressure and the remaining genome. We focused on CpGs inside CpG islands and on the species Homo Sapiens. We chose a genome-wide approach using computational biology methods.

One of the difficulties in this kind of study concerns the methods to be used to detect signatures left by natural selection. Despite the many methods that have been developed, up to now no method could be considered the “best one”. Each method apparently provides distinct information about selective events [17]. To overcome this limit we decided to use three different approaches. The first one, the iHS score [23], is a population genetic approach. The general idea of this method is to search for haplotypes longer than expected, the so-called “long-range haplotype”. An allele under selection increases in frequency so rapidly that long-range associations with neighboring polymorphisms are not disrupted by recombination. Generally this approach is thought to provide evidence for recent positive pressure [23], “recent” meaning after the human population separation. The second method defines as “under selective pressure” the regions of the human genome with a strong signal for depletion of Neanderthal-derived alleles. The presence of these signals may mark an episode of positive selection in early humans, after the separation from Neanderthal [25]. The third and last method belongs to the comparative approaches, involving data from multiple different species. Methods for detecting signatures of selection from rates and patterns of substitution have a long history in the field of molecular evolution [6]. The method that we used [26] is aimed to identify conserved elements in primates allowing to test hypotheses about selective pressures on this particular evolutionary lineage. We decided to use these three methods because they provide information about selective events happened in different evolutionary times.

Independently of the method that we used, CGIs localized inside regions under selective pressure were less methylated than CGIs in other genomic regions. In addition, we found that CGIs in regions with two signatures of selective pressure (in which CE is involved) showed lower methylation when compared to CGIs in regions with one signature only. This finding suggests that each signature is providing distinct information about selective events.

We observed CGIs undermethylation in all cell lines analyzed, including different types of normal cultured cells (fibroblasts, epithelial cells, myocytes etc.). It is well known that, in a multicellular organism, different cell types acquire various functional capabilities by distinct epigenetic modifications. Acquired during early development, the cell type-specific epigenotype is maintained by cellular memory mechanisms. It is quite surprising that different cells showed similar methylation differences. This finding may suggest that the regions under selective pressure are somehow more “protected” from methylation, independently of the cell type-specific epigenotype. This interpretation could be further supported by the analysis of EBV transformed and cancer derived cells. Epigenetics of cancer has been deeply studied, and the loss of DNA methylation at CpG dinucleotides was the first epigenetic abnormality to be identified in cancer cells [30]. The role of hypomethylation in activating oncogenes, as well as hypermethylation affects tumor-suppressor genes has been well established [30]. We found that genomic regions under selective pressure are relatively less methylated in cancer cells too. This difference persists even in a scenario of global hypermethylation that characterizes cancer cells in our experiments.

To confirm the results obtained in cell lines, we checked the possible existence of undermethylation in regions under selective pressure by a different approach. It is well established in scientific literature that the 5methylcytosine present in some CpG sites is subject to mutational pressure by spontaneous deamination to thymine [31]. A fraction of CpG sites in the genome are clustered into CpG islands that are thought to be mainly unmethylated [32]. Since 5-Methylcytosine is the initial molecule in the deamination reaction that generates thymine, CpG sequences within CpG islands, which are not methylated, would be less likely to mutate. Tomso et al. found a general underrepresentation of polymorphisms in CpG islands, strongly supporting the idea that decreased methylation in CpG islands leads to decreased variation at island CpGs [33]. Using the same way of reasoning, we predicted that, if CGIs in regions under selective pressure were undermethylated, they would show less polymorphisms than the CGIs in the remaining genome.

Independently of the method used to define the regions under selective pressure, we found that CGIs inside regions under selective pressure contain less SNPs than the CGIs in the remaining genome. When we compared CGIs in regions with two signatures of selective pressure to CGIs in regions with one signature only, we found that CGIs showing both 5SLR and CE signatures contained less SNPs than CGIs showing CE signature only. On the contrary, when we compared CGIs showing both HIR and CE signatures to CGIs showing CE signature only, we found no differences in SNP content. A possible explanation is that the selective pressure that acted on HIRs was very recent. Its effect could be evident in cell CGIs methylation but not (or not yet) in genetic variation.

CGIs can be located inside the genes or outside them. CGIs located inside genes can be divided, according their position, in CGIs in 5′ region, CGIs in the 3′ regions and CGIs in internal exons or introns. CGIs located near 5′ region of genes are known to influence gene expression but also CGIs located outside these regions can be involved in important biological processes [3] [34] [35]. We decided to analyze the methylation of CGIs, categorized by their position, to assess if the CGIs undermethylation that we have found in regions under selective pressure was a general phenomenon or it was restricted to CGIs with a specific localization. We were able to analyze only CE CGIs because, after classification, the number of HIR CGIs and 5LSR CGIs in intragenic and 3′ regions was too low to perform a reliable statistical analysis. We found that, at least for CE, the CGIs undermethylation in regions under selective pressure specifically involved CGIs located at the 5′ and in the intergenic regions. For the 5′ regions, the finding was quite expected because of their well established role in gene regulation. The functional role of intergenic CGIs is less clear. There is a growing evidence of the role of CGIs methylation in the regulation of microRNAs [36]. In particular, it has been demonstrated that 80% of the promoters of “intergenic” microRNAs contain CGIs. In addition, these regulatory regions show signals of evolutionary conservation [37]. We also cannot exclude that some CGIs categorized as intergenic, may be related to yet unidentified genes.

Bock et al. developed a computational epigenetics approach to discriminate between CpG islands that are prone to methylation from those that remain unmethylated on the basis of a set of 1,184 DNA attributes [12] . One of these attributes was the evolutionary conservation that the authors found to be uncorrelated with CpG island methylation. It should be noted that in this study (published in 2006) only CGIs on chromosome 21 were analyzed. Further, the methods to evaluate evolutionary conservation and for the statistical analysis are not the same that we used.

Our study has some limit. The most important one is the estimation of CGIs methylation. For each CGI we have data only on a limited number of CpGs, and from their methylation values we estimated the total CGI methylation. It should be noted that the dataset that we used is the largest genome-wide dataset available and that, in any case, this could be considered a systematic error that could cause a general noise only.

Another limit is that we analyzed the DNA methylation only. Epigenetic control of transcription involves a complex network of signals, including transcription factors, noncoding RNAs, DNA methylation, and histone modifications [38]. In this study we looked only to a part of these mechanisms. Further studies are needed to analyze the other component of this machinery.

Another possible limit concerns the method used to define regions under selective pressure. Other methods have been described and our choice could not be exhaustive. A final caveat concerns possible cell-culture induced DNA methylation. It is well established that in vitro culture can cause changes in epigenetic marking of the genome [39] [40], probably due to the adaptation of the cells to the in vitro conditions. Therefore it should be underlined that, concerning DNA methylation, cell lines could be not representative of their relative primary tissues.

In conclusion, in this paper we demonstrated, in several cell lines, that CpG islands in regions showing signatures of selective pressure are undermethylated in comparison with the other regions of the genome. Additionally, by analyzing SNP frequency in CpG islands, we demonstrated that CpG islands in regions under selective pressure show lower genetic variation among individuals.

## Materials and Methods

### Data and evolutionary scores

All the data and the scores that we used were downloaded from annotation tracks in the UCSC Genome Browser [41] . A brief description is provided below. Further and more detailed information about the dataset used can be found at http://genome.ucsc.edu/.

#### CpG island coordinates

CGIs genomic coordinates were obtained from the UCSC GB CpgIslandExt track. In this track CpG islands were predicted by searching the sequence one base at a time, scoring each dinucleotide (+17 for CG and −1 for others) and identifying maximally scoring segments. In this dataset, to define a CpG island the following criteria were used: i) to have a GC content of 50% or greater, ii) to have a length greater than 200 bp, and iii) to show a ratio greater than 0.6 of observed number of CG dinucleotides to the expected number, calculated on the basis of the number of Gs and Cs in the segment under analysis.

#### DNA methylation data

Methylation profiles from each cell sample were downloaded from the UCSC GB HAIB Methyl RRBS Track. These tables report the percentage of DNA molecules that show cytosine methylation at specific CpG dinucleotides in several cell lines. To obtain these data, researchers belonging the ENCODE Consortium assayed DNA methylation at CpG sites with a modified version of Reduced Representation Bisulfite Sequencing [20]. We used data from 25 cell lines, which were the first ones to come out from the moratorium period (expiration of moratorium period = 2011-04-13). The data set contains, for each cell line at least two replicas each containing, on average, about 1.5 million of CpG methylation values. To exclude unreliable data, only methylation signals identified by a number of reads ≥10 were used for further analyses. After this filtering, we computed, for each CpG the mean value between two replicas, obtaining methylation values of genomic CpGs per cell line in the range (5–8) 10^5^ . We next selected methylation values of CpG dinucleotides in CGIs, filtering them by the CpG Islands track of UCSC-GB. The final CGI methylation value was obtained by calculating the mean methylation of all CpGs contained in the CGI.

#### Integrated haplotype score (iHS)

The normalized iHS scores were obtained from UCSC Genome Browser “HGDP iHS” track. The per-continent integrated haplotype score (iHS) [23] is a measure of recent positive selection. The scores present in the UCSC Genome Browser were calculated using SNPs genotyped in 53 populations worldwide by the Human Genome Diversity Project in collaboration with the Centre d'Etude du Polymorphisme Humain (HGDP-CEPH).

Samples from 1,043 individuals from different geographical regions were genotyped for 657,000 SNPs at Stanford. The 53 populations were divided into seven continental groups: Africa (Bantu populations only), Middle East, Europe, South Asia, East Asia, Oceania and the Americas.

iHS was calculated for each population group and then normalizing the resulting unstandardized iHS scores in derived allele frequency bins as described in [23]. Per-SNP iHS scores were smoothed in windows of 31 SNPs, centered on each SNP. The final score is −log10 of the proportion of smoothed scores higher than each SNP's smoothed score.

We converted genome coordinates from assembly NCBI36/hg18 to assembly GRCh37/hg19 by using Batch Coordinate Conversion (liftOver) utility (UCSC Genome Browser). We scanned normalized iHS scores across the whole genome and selected the genomic intervals where iHS values ≥2. Once detected such compact regions, we extended their boundaries to the nearest loci where iHS was exactly vanishing.

#### Selective Sweep Scan: 5% Smallest S scores

Green et al. [25] identified polymorphic sites among five modern human genomes and determined ancestral or derived state of each single SNP. The human allele states were used to estimate an expected number of derived alleles in Neanderthal in the 100,000-base window around each SNP. The measure called S score compare the observed number of Neanderthal alleles in each window to the expected number. An S score significantly less than zero indicates an increase of human-derived alleles not found in Neanderthal, suggesting positive selection in the human lineage since divergence from Neanderthals.

Regions with S scores in the lowest 5% (strongest negative scores, “5% Lowest S” track of UCSC Genome Browser) were used in our analyses.

#### Conserved Elements

Conserved elements were downloaded from the UCSC GB Conservation (cons46way) Track. In this track conserved elements were predicted using the methods phastCons and phyloP. Both phastCons and phyloP are phylogenetic methods that rely on a tree model containing the tree topology, branch lengths representing evolutionary distance at neutrally evolving sites, the background distribution of nucleotides, and a substitution rate matrix. Pairwise alignments with the human genome were generated for each species using blastz from repeat-masked genomic sequence. The conserved elements were predicted using 10 primate species. Primate species used are :Homo Sapiens (reference species), Pan troglodytes, Gorilla gorilla gorilla, Pongo pygmaeus abelii, Macaca mulatta, Papio hamadryas, Callithrix jacchus, Tarsier syrichta, Microcebus murinus, Otolemur garnettii.

### Statistical analysis

In order to test the null hypothesis that two distributions have the same means we use a “bootstrapping approach”. In particular we take the mean of the smaller sample, hereafter denoted by μ, and compare this value with the probability distribution of mean, p(m), obtained from a large number (10^4^) of randomly sampled cohorts of the same size taken from the larger sample. Type I error to reject the null hypothesis even if it is true, denoted as “Bootstrap p-value” of the test, by definition is the sum of p(m) for m≥μ. Since we have 10^4^ cohorts of the larger sample the precision of our “Bootstrap p-value” is 10^−4^, which is however small enough since we have fixed the threshold of statistical significance at 10^−3^. All statistical analyses were carried out with R ver. 2.10.1 [42]

## Supporting Information

Figure S1 Histogram of CGIs mean methylation values (y-axis) and their Standard Errors for each cell line group: Cancer Transformed (CT), EBV transformed (EBV), and Normal Untransformed (NU).
(TIF)

Figure S2 Histogram of the percentages of methylation of HIR+CE CGIs (open bars) compared to CE CGIs (closed bars) for each cell line. Error bars represent standard errors.
(TIF)

Figure S3 Histogram of the percentages of methylation of 5SLR+CE CGIs (open bars) compared to CE CGIs (closed bars) for each cell line. Error bars represent standard errors.
(TIF)

Table S1 Complete list of the cell used in this study, with their characteristics.
(DOC)

Table S2 Lists, for each cell type, the number of CpG analyzed, the number of CpGs inside CGIs, the number of CGIs for which we were able to estimate methylation, the number of CpG analyzed per CGI and the mean value of CGI methylation.
(DOC)

Table S3 Lists, for each HIR identified, the chromosome, the start position, the end position, the total length and the human population in which it has been detected. Genomic coordinates refer to assembly GRCh37/hg19.
(DOC)

Table S4 Lists, for each cell type, the mean methylation of CGIs inside HIRs (with its standard error), the mean methylation of CGIs localized outside these regions (with its standard error), the number of CGIs inside HIRs, the number of CGIs localized outside HIRs and the Bootstrap p-values.
(DOC)

Table S5 Lists, for each 5SLR identified, the chromosome, the start position, the end position and the total length. Genomic coordinates refer to assembly GRCh37/hg19.
(DOC)

Table S6 Lists, for each cell type, the mean methylation of CGIs inside 5SLRs (with its standard error), the mean methylation of CGIs localized outside these regions (with its standard error), the number of CGIs inside 5SLRs s, the number of CGIs localized outside 5SLRs and the Bootstrap p-values.
(DOC)

Table S7 Lists, for each cell type, the mean methylation of CGIs containing CEs (with its standard error), the mean methylation of CGIs that not contain CEs (with its standard error), the number of CE CGIs, the number of non-CE CGIs and the Bootstrap p-values.
(DOC)

Table S8 Lists, for each cell type, the mean methylation of HIR+CE CGIs (with its standard error), the mean methylation of CE CGIs (with its standard error), the number of HIR+CE CGIs, the number of CE CGIs and the Bootstrap p-values.
(DOC)

Table S9 Lists, for each cell type, the mean methylation of 5SLR+CE CGIs (with its standard error), the mean methylation of CE CGIs (with its standard error), the number of 5SLR+CE CGIs, the number of CE CGIs and the Bootstrap p-values.
(DOC)

Table S10 Lists, for each cell type, the number, the mean methylation and the standard error of 5′ CGIs, intragenic CGIs, 3′ CGIs and intergenic CGIs.
(DOC)

Table S11 Lists, for each cell type and for each CGIs class (5′ CGIs, intragenic CGIs, 3′ CGIs and intergenic CGIs) the number and the mean methylation of CGIs containing CEs (with its standard error), the number and the mean methylation of CGIs that do not contain CEs (with its standard error), and the Bootstrap p-values.
(DOC)
