# Supplementary material for: CpG Islands Undermethylation in Human Genomic Regions under Selective Pressure
Source: PLoS One. 2011 Aug 2;6(8):e23156. doi: 10.1371/journal.pone.0023156 (PMC3149076; doi:10.1371/journal.pone.0023156)
Supplement: Table S2 — Lists, for each cell type, the number of CpG analyzed, the number of CpGs inside CGIs, the number of CGIs for which we were able to estimate methylation, the number of CpG analyzed per CGI and the mean value of CGI methylation. (DOC) [file pone.0023156.s005.doc]

| **Cell line** | **Total no. of CpG analyzed** | **No. of CpGs inside the CGIs** | **No. of CGIs with estimated methylation** | **No. of CpG analyzed/CGI** | **Mean value of CGI methylation** |
| --- | --- | --- | --- | --- | --- |
| **Hek293** | 556343 | 410965 | 16821 | 24.43 | 21.35 |
| **MCF-7** | 691911 | 477299 | 18199 | 26.23 | 32.77 |
| **Hepg2** | 632281 | 445920 | 18191 | 24.51 | 27.02 |
| **Cmk** | 668036 | 469564 | 17843 | 26.32 | 32.93 |
| **NB4** | 671651 | 482994 | 17845 | 27.07 | 31.47 |
| **NT2-D1** | 546153 | 414540 | 16471 | 25.17 | 15.95 |
| **Gm19239** | 520138 | 374026 | 16858 | 22.19 | 17.47 |
| **Gm19240** | 683988 | 476468 | 18577 | 25.65 | 20.39 |
| **Ag04449** | 601041 | 467048 | 17108 | 27.30 | 9.96 |
| **Ag04450** | 630183 | 447284 | 18043 | 24.79 | 13.66 |
| **Ag09309** | 691076 | 493539 | 18113 | 27.25 | 16.79 |
| **Ag09319** | 545847 | 396283 | 17150 | 23.11 | 14.44 |
| **Ag10803** | 774389 | 535976 | 19161 | 27.97 | 15.66 |
| **Fibrobl** | 632283 | 451557 | 17608 | 25.64 | 17.17 |
| **HAEpiC** | 681081 | 495882 | 18034 | 27.50 | 13.75 |
| **HCF** | 512497 | 378981 | 16137 | 23.49 | 12.6 |
| **HCM** | 760148 | 548934 | 18646 | 29.44 | 13.29 |
| **HEEpiC** | 624594 | 449729 | 18644 | 24.12 | 13.06 |
| **HIPEpiC** | 628948 | 459947 | 17722 | 25.95 | 13.22 |
| **HMEC** | 635871 | 460372 | 17825 | 25.83 | 16 |
| **HNPCEpiC** | 718881 | 530483 | 18431 | 28.78 | 12.8 |
| **HRCEpiC** | 505006 | 374743 | 16807 | 22.30 | 11.72 |
| **HSMMtube** | 704265 | 493549 | 14869 | 33.19 | 19.48 |
| **NHBE** | 680749 | 491581 | 18156 | 27.08 | 14.18 |
| **Skmc** | 674758 | 477801 | 16471 | 29.01 | 15.95 |
