# Supplementary material for: CpG Islands Undermethylation in Human Genomic Regions under Selective Pressure
Source: PLoS One. 2011 Aug 2;6(8):e23156. doi: 10.1371/journal.pone.0023156 (PMC3149076; doi:10.1371/journal.pone.0023156)
Supplement: Table S3 — Lists, for each HIR identified, the chromosome, the start position, the end position, the total length and the human population in which it has been detected. Genomic coordinates refer to assembly GRCh37/hg19. (DOC) [file pone.0023156.s006.doc]

| **Chromosome** | **Start** | **End** | **Length** | **Population** |
| --- | --- | --- | --- | --- |
| chr1 | 202566430 | 202843126 | 276696 | Bantu |
| chr1 | 113828836 | 114587765 | 758929 | Bantu |
| chr1 | 175113118 | 175336529 | 223411 | Bantu |
| chr1 | 160838638 | 161232771 | 394133 | Bantu |
| chr1 | 37023791 | 37307280 | 283489 | Bantu |
| chr1 | 79217345 | 79519426 | 302081 | Bantu |
| chr1 | 88983612 | 89384716 | 401104 | Bantu |
| chr1 | 35322503 | 36756490 | 1433987 | Middle East |
| chr1 | 229851411 | 230250181 | 398770 | Middle East |
| chr1 | 155059851 | 156078248 | 1018397 | Middle East |
| chr1 | 74560411 | 75027257 | 466846 | Middle East |
| chr1 | 154795389 | 156148164 | 1352775 | East Asia |
| chr1 | 65661665 | 66225796 | 564131 | East Asia |
| chr1 | 8320045 | 9000881 | 680836 | East Asia |
| chr1 | 172498216 | 173266577 | 768361 | East Asia |
| chr1 | 234701860 | 234906828 | 204968 | East Asia |
| chr1 | 30348925 | 30562065 | 213140 | East Asia |
| chr1 | 75098453 | 76399205 | 1300752 | East Asia |
| chr1 | 23184206 | 23834485 | 650279 | East Asia |
| chr1 | 64163143 | 64490892 | 327749 | East Asia |
| chr1 | 76463072 | 76729910 | 266838 | East Asia |
| chr1 | 81474889 | 81908616 | 433727 | East Asia |
| chr1 | 92464663 | 93523082 | 1058419 | East Asia |
| chr1 | 234637347 | 234853058 | 215711 | South Asia |
| chr1 | 186205867 | 186700958 | 495091 | South Asia |
| chr1 | 219644224 | 220082149 | 437925 | South Asia |
| chr1 | 236708968 | 236993127 | 284159 | South Asia |
| chr1 | 248004775 | 248344597 | 339822 | Europe |
| chr1 | 35321242 | 36833079 | 1511837 | Europe |
| chr1 | 161950451 | 162303458 | 353007 | Europe |
| chr1 | 186164899 | 186719569 | 554670 | Europe |
| chr1 | 30638016 | 30791524 | 153508 | Europe |
| chr1 | 184905261 | 185437237 | 531976 | Europe |
| chr1 | 220535456 | 220934076 | 398620 | Europe |
| chr1 | 11419868 | 11736599 | 316731 | Europe |
| chr1 | 89415576 | 90132068 | 716492 | America |
| chr1 | 161263380 | 162008078 | 744698 | America |
| chr2 | 155649338 | 156309902 | 660564 | Bantu |
| chr2 | 72353138 | 73170729 | 817591 | Bantu |
| chr2 | 212010493 | 212199993 | 189500 | Bantu |
| chr2 | 238592234 | 238789194 | 196960 | Bantu |
| chr2 | 209141066 | 209434192 | 293126 | Bantu |
| chr2 | 33174806 | 33338735 | 163929 | Bantu |
| chr2 | 46622873 | 46748065 | 125192 | Bantu |
| chr2 | 140962703 | 141212782 | 250079 | Bantu |
| chr2 | 200751772 | 201256573 | 504801 | Bantu |
| chr2 | 72239692 | 73220137 | 980445 | Middle East |
| chr2 | 1752229 | 2163075 | 410846 | Middle East |
| chr2 | 178148204 | 178579812 | 431608 | Middle East |
| chr2 | 195377437 | 195968049 | 590612 | Middle East |
| chr2 | 14756349 | 15076200 | 319851 | Middle East |
| chr2 | 74463566 | 75012326 | 548760 | Middle East |
| chr2 | 235709572 | 235971970 | 262398 | Middle East |
| chr2 | 21587225 | 22197491 | 610266 | Middle East |
| chr2 | 117838706 | 118274001 | 435295 | Middle East |
| chr2 | 157923137 | 158915734 | 992597 | Middle East |
| chr2 | 108546122 | 109825515 | 1279393 | East Asia |
| chr2 | 16798475 | 18056446 | 1257971 | East Asia |
| chr2 | 212788895 | 213695701 | 906806 | East Asia |
| chr2 | 84536960 | 85288571 | 751611 | East Asia |
| chr2 | 125232609 | 127372727 | 2140118 | East Asia |
| chr2 | 154510972 | 155053139 | 542167 | East Asia |
| chr2 | 8794240 | 9870630 | 1076390 | East Asia |
| chr2 | 177293076 | 179236047 | 1942971 | East Asia |
| chr2 | 223503370 | 223856275 | 352905 | East Asia |
| chr2 | 43638712 | 44129773 | 491061 | East Asia |
| chr2 | 177914806 | 178612845 | 698039 | South Asia |
| chr2 | 231638974 | 231869040 | 230066 | South Asia |
| chr2 | 195552753 | 197098637 | 1545884 | South Asia |
| chr2 | 223549297 | 224218792 | 669495 | South Asia |
| chr2 | 157993888 | 158922574 | 928686 | South Asia |
| chr2 | 82644948 | 83438063 | 793115 | South Asia |
| chr2 | 73590806 | 74119507 | 528701 | South Asia |
| chr2 | 178158043 | 178612269 | 454226 | Europe |
| chr2 | 167525345 | 168461160 | 935815 | Europe |
| chr2 | 197255486 | 198056040 | 800554 | Europe |
| chr2 | 195198744 | 196465135 | 1266391 | Europe |
| chr2 | 74461634 | 75008283 | 546649 | Europe |
| chr2 | 196622252 | 197143630 | 521378 | Europe |
| chr2 | 158095057 | 158890761 | 795704 | Europe |
| chr2 | 121152814 | 121332190 | 179376 | Europe |
| chr2 | 141536876 | 141803601 | 266725 | Europe |
| chr2 | 17432556 | 18185589 | 753033 | America |
| chr2 | 169610232 | 169870854 | 260622 | America |
| chr2 | 36045002 | 36399317 | 354315 | America |
| chr2 | 39776534 | 40283458 | 506924 | America |
| chr2 | 102288186 | 103208609 | 920423 | America |
| chr3 | 4445436 | 4593187 | 147751 | Bantu |
| chr3 | 29582412 | 29735362 | 152950 | Bantu |
| chr3 | 56467691 | 56858493 | 390802 | Bantu |
| chr3 | 61066987 | 61267415 | 200428 | Bantu |
| chr3 | 76685884 | 76869830 | 183946 | Bantu |
| chr3 | 30263217 | 30416461 | 153244 | Bantu |
| chr3 | 63029516 | 63168593 | 139077 | Bantu |
| chr3 | 105006914 | 105670159 | 663245 | Bantu |
| chr3 | 25495637 | 26448193 | 952556 | Middle East |
| chr3 | 5547942 | 5777221 | 229279 | Middle East |
| chr3 | 148640368 | 148947187 | 306819 | Middle East |
| chr3 | 175042776 | 175530096 | 487320 | East Asia |
| chr3 | 104618616 | 104903304 | 284688 | East Asia |
| chr3 | 44091635 | 45170504 | 1078869 | East Asia |
| chr3 | 195606770 | 196351214 | 744444 | East Asia |
| chr3 | 25666156 | 26450380 | 784224 | East Asia |
| chr3 | 26976847 | 27638499 | 661652 | East Asia |
| chr3 | 112911525 | 113233187 | 321662 | East Asia |
| chr3 | 166170696 | 167890717 | 1720021 | East Asia |
| chr3 | 25489318 | 26447274 | 957956 | South Asia |
| chr3 | 140485038 | 141050804 | 565766 | South Asia |
| chr3 | 129699722 | 130468624 | 768902 | South Asia |
| chr3 | 165267688 | 166012937 | 745249 | South Asia |
| chr3 | 12481375 | 12857492 | 376117 | South Asia |
| chr3 | 66744399 | 66996178 | 251779 | South Asia |
| chr3 | 101816344 | 102457117 | 640773 | South Asia |
| chr3 | 182957573 | 183177706 | 220133 | South Asia |
| chr3 | 72533768 | 72667610 | 133842 | Europe |
| chr3 | 129658706 | 130477565 | 818859 | Europe |
| chr3 | 190295730 | 190629384 | 333654 | Europe |
| chr3 | 156259485 | 156821807 | 562322 | America |
| chr3 | 100561439 | 100892053 | 330614 | America |
| chr3 | 20984501 | 21456169 | 471668 | America |
| chr3 | 63663692 | 64099568 | 435876 | America |
| chr4 | 118796265 | 119853637 | 1057372 | Bantu |
| chr4 | 41798793 | 42165798 | 367005 | Bantu |
| chr4 | 129779076 | 130144490 | 365414 | Bantu |
| chr4 | 33653757 | 35047016 | 1393259 | Bantu |
| chr4 | 65507693 | 65895584 | 387891 | Bantu |
| chr4 | 429720 | 871674 | 441954 | Bantu |
| chr4 | 86088554 | 86358620 | 270066 | Bantu |
| chr4 | 111540391 | 111765494 | 225103 | Bantu |
| chr4 | 33756410 | 34855431 | 1099021 | Middle East |
| chr4 | 169771612 | 170787253 | 1015641 | Middle East |
| chr4 | 148032003 | 148987971 | 955968 | Middle East |
| chr4 | 14442433 | 14861269 | 418836 | Middle East |
| chr4 | 29775276 | 30429541 | 654265 | Middle East |
| chr4 | 41322398 | 42206573 | 884175 | Middle East |
| chr4 | 172311051 | 172742576 | 431525 | Middle East |
| chr4 | 104890866 | 105457179 | 566313 | East Asia |
| chr4 | 143493213 | 144542212 | 1048999 | East Asia |
| chr4 | 152792791 | 153097095 | 304304 | East Asia |
| chr4 | 157656264 | 160680412 | 3024148 | East Asia |
| chr4 | 41322398 | 42244444 | 922046 | East Asia |
| chr4 | 169669891 | 170762016 | 1092125 | East Asia |
| chr4 | 41407668 | 42241035 | 833367 | South Asia |
| chr4 | 5171986 | 5426326 | 254340 | South Asia |
| chr4 | 173213263 | 173863771 | 650508 | South Asia |
| chr4 | 179935508 | 180172955 | 237447 | South Asia |
| chr4 | 32672332 | 34855431 | 2183099 | Europe |
| chr4 | 5218136 | 5425361 | 207225 | Europe |
| chr4 | 41333429 | 42227496 | 894067 | Europe |
| chr4 | 171743216 | 172783333 | 1040117 | Europe |
| chr4 | 148025559 | 148491479 | 465920 | Europe |
| chr4 | 14438997 | 14862872 | 423875 | Europe |
| chr4 | 123228113 | 124245085 | 1016972 | Europe |
| chr4 | 42331579 | 42627835 | 296256 | America |
| chr4 | 126738833 | 127236804 | 497971 | America |
| chr4 | 128495654 | 129509264 | 1013610 | America |
| chr5 | 108964907 | 109272681 | 307774 | Bantu |
| chr5 | 160174288 | 160380400 | 206112 | Bantu |
| chr5 | 66794632 | 67121200 | 326568 | Bantu |
| chr5 | 87125578 | 88021526 | 895948 | Bantu |
| chr5 | 169485227 | 169625918 | 140691 | Bantu |
| chr5 | 25495542 | 25691581 | 196039 | Bantu |
| chr5 | 142031751 | 142520585 | 488834 | Middle East |
| chr5 | 24497516 | 24847070 | 349554 | Middle East |
| chr5 | 11366401 | 11893756 | 527355 | Middle East |
| chr5 | 19493684 | 20429331 | 935647 | Middle East |
| chr5 | 109401940 | 110455898 | 1053958 | Middle East |
| chr5 | 30028447 | 30913502 | 885055 | Middle East |
| chr5 | 92219182 | 92675984 | 456802 | Middle East |
| chr5 | 90466296 | 90782146 | 315850 | Middle East |
| chr5 | 37913527 | 38135717 | 222190 | East Asia |
| chr5 | 97821452 | 98569708 | 748256 | East Asia |
| chr5 | 107994013 | 108579888 | 585875 | East Asia |
| chr5 | 113425033 | 113847911 | 422878 | East Asia |
| chr5 | 172845597 | 173103336 | 257739 | East Asia |
| chr5 | 92595749 | 93637332 | 1041583 | East Asia |
| chr5 | 54601452 | 55204186 | 602734 | South Asia |
| chr5 | 109636606 | 110500986 | 864380 | South Asia |
| chr5 | 37909151 | 38166348 | 257197 | South Asia |
| chr5 | 158944085 | 159230739 | 286654 | South Asia |
| chr5 | 120960853 | 121265924 | 305071 | South Asia |
| chr5 | 142031751 | 142238118 | 206367 | South Asia |
| chr5 | 165886820 | 166090938 | 204118 | South Asia |
| chr5 | 108031617 | 108581017 | 549400 | South Asia |
| chr5 | 109475723 | 110399880 | 924157 | Europe |
| chr5 | 21541191 | 22083472 | 542281 | Europe |
| chr5 | 37915147 | 38165207 | 250060 | Europe |
| chr5 | 24486805 | 24851079 | 364274 | Europe |
| chr5 | 142020380 | 142529959 | 509579 | Europe |
| chr5 | 80687791 | 81327041 | 639250 | America |
| chr5 | 140553664 | 141326961 | 773297 | America |
| chr5 | 164283543 | 164865385 | 581842 | America |
| chr5 | 59556461 | 60224913 | 668452 | America |
| chr5 | 153277759 | 153988723 | 710964 | America |
| chr6 | 74981125 | 75538209 | 557084 | Bantu |
| chr6 | 76857008 | 77299932 | 442924 | Bantu |
| chr6 | 130503306 | 130789540 | 286234 | Bantu |
| chr6 | 72662228 | 73018440 | 356212 | Bantu |
| chr6 | 168410476 | 168546851 | 136375 | Bantu |
| chr6 | 40420901 | 40616865 | 195964 | Bantu |
| chr6 | 70802259 | 70990469 | 188210 | Bantu |
| chr6 | 132919948 | 133067732 | 147784 | Bantu |
| chr6 | 97538657 | 98044631 | 505974 | Bantu |
| chr6 | 73650662 | 73866941 | 216279 | Middle East |
| chr6 | 107420270 | 108089687 | 669417 | Middle East |
| chr6 | 1045539 | 1149482 | 103943 | Middle East |
| chr6 | 4744063 | 4984230 | 240167 | Middle East |
| chr6 | 14688469 | 15016186 | 327717 | Middle East |
| chr6 | 16109163 | 16362387 | 253224 | Middle East |
| chr6 | 123902541 | 124158251 | 255710 | Middle East |
| chr6 | 25352637 | 27356924 | 2004287 | East Asia |
| chr6 | 105369510 | 106017975 | 648465 | East Asia |
| chr6 | 37278933 | 37585912 | 306979 | East Asia |
| chr6 | 73650662 | 74064395 | 413733 | South Asia |
| chr6 | 105105089 | 105977159 | 872070 | South Asia |
| chr6 | 213983 | 412740 | 198757 | South Asia |
| chr6 | 104765922 | 106006229 | 1240307 | Europe |
| chr6 | 73652443 | 73893196 | 240753 | Europe |
| chr6 | 167169223 | 167521623 | 352400 | Europe |
| chr6 | 121812866 | 123188087 | 1375221 | America |
| chr6 | 46859043 | 47196209 | 337166 | America |
| chr6 | 46283922 | 46745998 | 462076 | America |
| chr6 | 152774033 | 153103098 | 329065 | America |
| chr6 | 158382074 | 158865656 | 483582 | America |
| chr6 | 33889404 | 36757265 | 2867861 | America |
| chr6 | 49902734 | 50629153 | 726419 | America |
| chr6 | 72806978 | 73161392 | 354414 | America |
| chr6 | 150748277 | 150945214 | 196937 | America |
| chr7 | 40485949 | 40946512 | 460563 | Bantu |
| chr7 | 2739017 | 2966137 | 227120 | Bantu |
| chr7 | 141214012 | 141710898 | 496886 | Bantu |
| chr7 | 28753387 | 28926167 | 172780 | Bantu |
| chr7 | 91998495 | 92480883 | 482388 | Bantu |
| chr7 | 17912336 | 18094286 | 181950 | Bantu |
| chr7 | 115654075 | 115851980 | 197905 | Bantu |
| chr7 | 123656664 | 124152389 | 495725 | Bantu |
| chr7 | 156416220 | 156754980 | 338760 | Bantu |
| chr7 | 118610284 | 120731948 | 2121664 | Middle East |
| chr7 | 124182414 | 124827235 | 644821 | Middle East |
| chr7 | 33555119 | 33839113 | 283994 | Middle East |
| chr7 | 97626161 | 98123879 | 497718 | Middle East |
| chr7 | 98772131 | 99447045 | 674914 | Middle East |
| chr7 | 3726122 | 4333609 | 607487 | East Asia |
| chr7 | 49640912 | 50313351 | 672439 | East Asia |
| chr7 | 28693593 | 28934502 | 240909 | East Asia |
| chr7 | 29833587 | 30217699 | 384112 | East Asia |
| chr7 | 64807261 | 66384314 | 1577053 | East Asia |
| chr7 | 111811004 | 112304166 | 493162 | East Asia |
| chr7 | 126457779 | 127852064 | 1394285 | East Asia |
| chr7 | 138518783 | 138794878 | 276095 | East Asia |
| chr7 | 117496319 | 120683750 | 3187431 | South Asia |
| chr7 | 36969211 | 37341476 | 372265 | South Asia |
| chr7 | 148636850 | 149017787 | 380937 | South Asia |
| chr7 | 36818523 | 37260711 | 442188 | Europe |
| chr7 | 98759117 | 99525240 | 766123 | Europe |
| chr7 | 119301786 | 120683750 | 1381964 | Europe |
| chr7 | 102486254 | 103231289 | 745035 | Europe |
| chr7 | 107948721 | 108262713 | 313992 | America |
| chr7 | 99300758 | 100968362 | 1667604 | America |
| chr7 | 122627567 | 123037886 | 410319 | America |
| chr7 | 92623141 | 93013573 | 390432 | America |
| chr8 | 21164751 | 21311469 | 146718 | Bantu |
| chr8 | 113645627 | 114021483 | 375856 | Bantu |
| chr8 | 9410495 | 9681256 | 270761 | Bantu |
| chr8 | 68653516 | 68838275 | 184759 | Bantu |
| chr8 | 99766557 | 100855842 | 1089285 | Bantu |
| chr8 | 5225481 | 5409370 | 183889 | Bantu |
| chr8 | 9087278 | 9243427 | 156149 | Bantu |
| chr8 | 139901178 | 140127522 | 226344 | Middle East |
| chr8 | 18514100 | 18673191 | 159091 | Middle East |
| chr8 | 59793692 | 59962803 | 169111 | Middle East |
| chr8 | 36629798 | 37046811 | 417013 | Middle East |
| chr8 | 129503361 | 130268559 | 765198 | East Asia |
| chr8 | 134804170 | 135328581 | 524411 | East Asia |
| chr8 | 21616454 | 21988514 | 372060 | South Asia |
| chr8 | 57826145 | 58251654 | 425509 | South Asia |
| chr8 | 66555818 | 67019987 | 464169 | South Asia |
| chr8 | 42045655 | 43215239 | 1169584 | South Asia |
| chr8 | 139926589 | 140131393 | 204804 | Europe |
| chr8 | 29692897 | 30218244 | 525347 | Europe |
| chr8 | 18511097 | 18673191 | 162094 | Europe |
| chr8 | 32507149 | 33806909 | 1299760 | America |
| chr8 | 99383408 | 100997713 | 1614305 | America |
| chr8 | 95303456 | 95847149 | 543693 | America |
| chr8 | 707884 | 1099985 | 392101 | America |
| chr9 | 23785967 | 24654574 | 868607 | Bantu |
| chr9 | 115404746 | 115678032 | 273286 | Bantu |
| chr9 | 114868003 | 115347087 | 479084 | Bantu |
| chr9 | 100507159 | 100871716 | 364557 | Bantu |
| chr9 | 3862693 | 3961491 | 98798 | Bantu |
| chr9 | 95374368 | 95855099 | 480731 | Bantu |
| chr9 | 96447721 | 96681387 | 233666 | Bantu |
| chr9 | 111693132 | 111961388 | 268256 | Bantu |
| chr9 | 76765949 | 77038958 | 273009 | Bantu |
| chr9 | 139647074 | 140376667 | 729593 | Bantu |
| chr9 | 8787733 | 8868683 | 80950 | Bantu |
| chr9 | 1707518 | 2165757 | 458239 | Middle East |
| chr9 | 107870014 | 108376435 | 506421 | Middle East |
| chr9 | 126154354 | 126768047 | 613693 | Middle East |
| chr9 | 12935290 | 13426560 | 491270 | Middle East |
| chr9 | 111537522 | 111961388 | 423866 | Middle East |
| chr9 | 136658987 | 136807769 | 148782 | Middle East |
| chr9 | 22986049 | 23425709 | 439660 | East Asia |
| chr9 | 126153636 | 126783846 | 630210 | East Asia |
| chr9 | 26506859 | 27148474 | 641615 | East Asia |
| chr9 | 106554028 | 106960541 | 406513 | East Asia |
| chr9 | 13780058 | 14014967 | 234909 | East Asia |
| chr9 | 111114252 | 111973019 | 858767 | East Asia |
| chr9 | 130755693 | 131580743 | 825050 | South Asia |
| chr9 | 1784090 | 2160104 | 376014 | South Asia |
| chr9 | 126154354 | 126769290 | 614936 | South Asia |
| chr9 | 107878896 | 108244575 | 365679 | South Asia |
| chr9 | 107869514 | 108315068 | 445554 | Europe |
| chr9 | 3161347 | 3579890 | 418543 | Europe |
| chr9 | 93928416 | 94448776 | 520360 | Europe |
| chr9 | 126154354 | 126749630 | 595276 | Europe |
| chr9 | 130908044 | 131595406 | 687362 | Europe |
| chr9 | 111639682 | 111957172 | 317490 | Europe |
| chr9 | 9729134 | 9893070 | 163936 | Europe |
| chr9 | 16046983 | 16252409 | 205426 | Europe |
| chr9 | 18944389 | 19144190 | 199801 | Europe |
| chr9 | 12925921 | 13755191 | 829270 | Europe |
| chr9 | 93946379 | 94498549 | 552170 | America |
| chr9 | 113300206 | 113527564 | 227358 | America |
| chr10 | 85960149 | 86356554 | 396405 | Bantu |
| chr10 | 90042154 | 90436200 | 394046 | Bantu |
| chr10 | 69021184 | 69430997 | 409813 | Bantu |
| chr10 | 100339741 | 101069351 | 729610 | Bantu |
| chr10 | 83577099 | 84424077 | 846978 | Middle East |
| chr10 | 118047298 | 118351904 | 304606 | Middle East |
| chr10 | 131166636 | 131498390 | 331754 | Middle East |
| chr10 | 21783634 | 22926992 | 1143358 | Middle East |
| chr10 | 53139938 | 53415187 | 275249 | East Asia |
| chr10 | 3877968 | 4469291 | 591323 | East Asia |
| chr10 | 107027076 | 107533730 | 506654 | East Asia |
| chr10 | 109428376 | 110348375 | 919999 | East Asia |
| chr10 | 73656982 | 74450318 | 793336 | East Asia |
| chr10 | 93348014 | 95233855 | 1885841 | East Asia |
| chr10 | 83583540 | 84386092 | 802552 | South Asia |
| chr10 | 59386322 | 60255968 | 869646 | South Asia |
| chr10 | 131160866 | 131494256 | 333390 | South Asia |
| chr10 | 100225657 | 101185829 | 960172 | South Asia |
| chr10 | 114912534 | 115198442 | 285908 | Europe |
| chr10 | 118052051 | 118381944 | 329893 | Europe |
| chr10 | 83835639 | 84386972 | 551333 | Europe |
| chr10 | 6768836 | 6930654 | 161818 | Europe |
| chr10 | 112570243 | 112918572 | 348329 | Europe |
| chr10 | 109950470 | 110410029 | 459559 | America |
| chr10 | 116565387 | 117735418 | 1170031 | America |
| chr11 | 110336182 | 110796084 | 459902 | Bantu |
| chr11 | 112727378 | 113103995 | 376617 | Bantu |
| chr11 | 9791579 | 10432369 | 640790 | Bantu |
| chr11 | 6043277 | 6301176 | 257899 | Bantu |
| chr11 | 129392269 | 129568491 | 176222 | Bantu |
| chr11 | 83638942 | 84024745 | 385803 | Middle East |
| chr11 | 129832270 | 130264277 | 432007 | Middle East |
| chr11 | 20702034 | 20979130 | 277096 | Middle East |
| chr11 | 12157657 | 12285089 | 127432 | Middle East |
| chr11 | 37977241 | 38407175 | 429934 | Middle East |
| chr11 | 24839058 | 25755820 | 916762 | East Asia |
| chr11 | 66688100 | 67438194 | 750094 | East Asia |
| chr11 | 80930312 | 81515166 | 584854 | East Asia |
| chr11 | 112812563 | 113056322 | 243759 | East Asia |
| chr11 | 87342854 | 87674034 | 331180 | South Asia |
| chr11 | 39439328 | 40043059 | 603731 | South Asia |
| chr11 | 129832270 | 130264277 | 432007 | South Asia |
| chr11 | 92108243 | 92623348 | 515105 | South Asia |
| chr11 | 105719522 | 106288553 | 569031 | South Asia |
| chr11 | 66240882 | 68468669 | 2227787 | South Asia |
| chr11 | 87187306 | 87678365 | 491059 | Europe |
| chr11 | 66687863 | 67414491 | 726628 | Europe |
| chr11 | 86403375 | 86586679 | 183304 | Europe |
| chr11 | 69231796 | 69579069 | 347273 | America |
| chr11 | 6577577 | 6885406 | 307829 | America |
| chr11 | 70966997 | 72935824 | 1968827 | America |
| chr11 | 42864284 | 43656534 | 792250 | America |
| chr12 | 28118847 | 28290777 | 171930 | Bantu |
| chr12 | 5374517 | 5555039 | 180522 | Bantu |
| chr12 | 81966423 | 82312036 | 345613 | Bantu |
| chr12 | 113458677 | 113935480 | 476803 | Bantu |
| chr12 | 126017498 | 126322479 | 304981 | Middle East |
| chr12 | 99265706 | 99870626 | 604920 | Middle East |
| chr12 | 10069302 | 10492981 | 423679 | Middle East |
| chr12 | 127043455 | 127329166 | 285711 | Middle East |
| chr12 | 121196891 | 121669181 | 472290 | Middle East |
| chr12 | 888428 | 1644884 | 756456 | East Asia |
| chr12 | 100097346 | 100868682 | 771336 | East Asia |
| chr12 | 24451627 | 24925238 | 473611 | East Asia |
| chr12 | 80462262 | 81032937 | 570675 | South Asia |
| chr12 | 99332599 | 99870626 | 538027 | South Asia |
| chr12 | 850613 | 1510504 | 659891 | South Asia |
| chr12 | 95184157 | 95735182 | 551025 | South Asia |
| chr12 | 59128693 | 59494105 | 365412 | South Asia |
| chr12 | 65827996 | 66140732 | 312736 | South Asia |
| chr12 | 2609562 | 2919700 | 310138 | Europe |
| chr12 | 11460063 | 11714653 | 254590 | Europe |
| chr12 | 10046052 | 10424626 | 378574 | Europe |
| chr12 | 126039433 | 126290696 | 251263 | Europe |
| chr12 | 99638276 | 99866158 | 227882 | Europe |
| chr12 | 102389496 | 102838514 | 449018 | Europe |
| chr12 | 47618366 | 48257765 | 639399 | America |
| chr12 | 3723673 | 4042533 | 318860 | America |
| chr13 | 30462712 | 30621645 | 158933 | Bantu |
| chr13 | 46755852 | 47132369 | 376517 | Bantu |
| chr13 | 104067224 | 104357021 | 289797 | Middle East |
| chr13 | 33191371 | 33553782 | 362411 | Middle East |
| chr13 | 74840712 | 75105119 | 264407 | Middle East |
| chr13 | 104989752 | 105265994 | 276242 | East Asia |
| chr13 | 34783249 | 35085471 | 302222 | East Asia |
| chr13 | 103992269 | 104361190 | 368921 | South Asia |
| chr13 | 67731496 | 68459217 | 727721 | South Asia |
| chr13 | 92250545 | 92847826 | 597281 | South Asia |
| chr13 | 45401128 | 45900266 | 499138 | South Asia |
| chr13 | 103989946 | 104354010 | 364064 | Europe |
| chr13 | 88900080 | 89934018 | 1033938 | America |
| chr13 | 38429073 | 38803973 | 374900 | America |
| chr14 | 60185336 | 61572948 | 1387612 | Bantu |
| chr14 | 98497054 | 98776337 | 279283 | Bantu |
| chr14 | 32007373 | 32391208 | 383835 | Bantu |
| chr14 | 43480483 | 44259508 | 779025 | Bantu |
| chr14 | 48570466 | 49066430 | 495964 | Bantu |
| chr14 | 33942839 | 34054942 | 112103 | Bantu |
| chr14 | 36524098 | 36809925 | 285827 | Bantu |
| chr14 | 72658681 | 72892393 | 233712 | Bantu |
| chr14 | 61662167 | 62159911 | 497744 | Middle East |
| chr14 | 100852820 | 101247031 | 394211 | Middle East |
| chr14 | 55218435 | 55888532 | 670097 | Middle East |
| chr14 | 87470029 | 87984703 | 514674 | East Asia |
| chr14 | 64873599 | 65660501 | 786902 | South Asia |
| chr14 | 62536948 | 64673866 | 2136918 | South Asia |
| chr14 | 102238630 | 102900767 | 662137 | South Asia |
| chr14 | 24406306 | 24903963 | 497657 | Europe |
| chr14 | 100852820 | 101240529 | 387709 | Europe |
| chr14 | 100781877 | 101200644 | 418767 | America |
| chr15 | 61994586 | 62397233 | 402647 | Bantu |
| chr15 | 50944589 | 51394410 | 449821 | Bantu |
| chr15 | 56337527 | 56659827 | 322300 | Bantu |
| chr15 | 95284161 | 95544856 | 260695 | Bantu |
| chr15 | 67049507 | 67234339 | 184832 | Middle East |
| chr15 | 69178312 | 69991416 | 813104 | Middle East |
| chr15 | 48132379 | 49038469 | 906090 | Middle East |
| chr15 | 93984201 | 94225522 | 241321 | Middle East |
| chr15 | 93293836 | 93648497 | 354661 | East Asia |
| chr15 | 67029307 | 67227210 | 197903 | South Asia |
| chr15 | 69424143 | 69991613 | 567470 | South Asia |
| chr15 | 91716130 | 91864817 | 148687 | South Asia |
| chr15 | 48224971 | 48812019 | 587048 | Europe |
| chr15 | 42744094 | 43796907 | 1052813 | Europe |
| chr15 | 72060049 | 73086730 | 1026681 | Europe |
| chr15 | 58210398 | 58601803 | 391405 | America |
| chr15 | 59655578 | 60363167 | 707589 | America |
| chr15 | 72299481 | 73109628 | 810147 | America |
| chr15 | 24267426 | 24999290 | 731864 | America |
| chr16 | 81365974 | 81595185 | 229211 | Bantu |
| chr16 | 22915924 | 23274063 | 358139 | Bantu |
| chr16 | 12655483 | 12704564 | 49081 | Bantu |
| chr16 | 25932733 | 26101005 | 168272 | Bantu |
| chr16 | 82987188 | 83162677 | 175489 | Middle East |
| chr16 | 1522670 | 2010137 | 487467 | Middle East |
| chr16 | 24609208 | 24888453 | 279245 | Middle East |
| chr16 | 65519567 | 65914253 | 394686 | East Asia |
| chr16 | 79679369 | 79981629 | 302260 | East Asia |
| chr16 | 17131750 | 17761858 | 630108 | East Asia |
| chr16 | 75492242 | 76018395 | 526153 | East Asia |
| chr16 | 79741569 | 79991174 | 249605 | Europe |
| chr16 | 82228076 | 82456634 | 228558 | Europe |
| chr16 | 10928112 | 11693535 | 765423 | America |
| chr16 | 13594921 | 13853466 | 258545 | America |
| chr16 | 82120192 | 82296370 | 176178 | America |
| chr16 | 11919959 | 12314450 | 394491 | America |
| chr16 | 70723925 | 73016767 | 2292842 | America |
| chr17 | 3423578 | 3769406 | 345828 | Bantu |
| chr17 | 45278790 | 45908404 | 629614 | Bantu |
| chr17 | 49929451 | 50258110 | 328659 | Bantu |
| chr17 | 19613601 | 20699544 | 1085943 | Bantu |
| chr17 | 57637803 | 59345378 | 1707575 | Middle East |
| chr17 | 55274630 | 55522449 | 247819 | Middle East |
| chr17 | 63123532 | 63541496 | 417964 | Middle East |
| chr17 | 74574103 | 74916503 | 342400 | Middle East |
| chr17 | 53573902 | 54158281 | 584379 | Middle East |
| chr17 | 76890864 | 77110043 | 219179 | Middle East |
| chr17 | 48367272 | 48860431 | 493159 | East Asia |
| chr17 | 27322441 | 29161357 | 1838916 | East Asia |
| chr17 | 57832391 | 59423419 | 1591028 | East Asia |
| chr17 | 63155764 | 63505321 | 349557 | South Asia |
| chr17 | 53563973 | 54094316 | 530343 | South Asia |
| chr17 | 63109056 | 63515782 | 406726 | Europe |
| chr17 | 53519844 | 54106536 | 586692 | Europe |
| chr17 | 74563175 | 74938080 | 374905 | Europe |
| chr17 | 9390389 | 9708955 | 318566 | Europe |
| chr17 | 76885117 | 77104322 | 219205 | Europe |
| chr17 | 38775805 | 39650803 | 874998 | America |
| chr17 | 35020291 | 35826181 | 805890 | America |
| chr18 | 42619149 | 42883958 | 264809 | Bantu |
| chr18 | 43959703 | 44146835 | 187132 | Bantu |
| chr18 | 37544937 | 37836985 | 292048 | Bantu |
| chr18 | 7384596 | 7706346 | 321750 | Middle East |
| chr18 | 66570423 | 66881411 | 310988 | Middle East |
| chr18 | 31042855 | 31383570 | 340715 | East Asia |
| chr18 | 66587607 | 66871598 | 283991 | South Asia |
| chr18 | 32009672 | 32375776 | 366104 | South Asia |
| chr18 | 57598173 | 57767634 | 169461 | South Asia |
| chr18 | 46076096 | 46240710 | 164614 | South Asia |
| chr18 | 7147079 | 7732916 | 585837 | Europe |
| chr18 | 66574279 | 66887310 | 313031 | Europe |
| chr19 | 51907788 | 52127052 | 219264 | Bantu |
| chr19 | 38602214 | 39002139 | 399925 | Bantu |
| chr19 | 42207625 | 43192390 | 984765 | Bantu |
| chr19 | 10621108 | 11163561 | 542453 | Bantu |
| chr19 | 31369317 | 31796288 | 426971 | Bantu |
| chr19 | 32083035 | 32471522 | 388487 | Bantu |
| chr19 | 36876843 | 37367131 | 490288 | Bantu |
| chr19 | 37412887 | 38475085 | 1062198 | Bantu |
| chr19 | 22635052 | 23430435 | 795383 | Middle East |
| chr19 | 22692592 | 23430435 | 737843 | South Asia |
| chr19 | 22709636 | 23461423 | 751787 | Europe |
| chr20 | 47177768 | 47591081 | 413313 | Bantu |
| chr20 | 37080007 | 37933672 | 853665 | Bantu |
| chr20 | 15017240 | 15178252 | 161012 | Bantu |
| chr20 | 52884386 | 53287736 | 403350 | Middle East |
| chr20 | 22282991 | 22703676 | 420685 | Middle East |
| chr20 | 15571727 | 15817105 | 245378 | Middle East |
| chr20 | 20993838 | 21906779 | 912941 | Middle East |
| chr20 | 49063166 | 49649709 | 586543 | Middle East |
| chr20 | 33737661 | 34875144 | 1137483 | East Asia |
| chr20 | 30437522 | 31712589 | 1275067 | East Asia |
| chr20 | 52804492 | 53288594 | 484102 | South Asia |
| chr20 | 22194770 | 22708358 | 513588 | South Asia |
| chr20 | 49097525 | 49653969 | 556444 | South Asia |
| chr20 | 53581724 | 53816637 | 234913 | South Asia |
| chr20 | 6893128 | 7164724 | 271596 | Europe |
| chr20 | 22185332 | 22506049 | 320717 | Europe |
| chr20 | 33844938 | 34442671 | 597733 | Europe |
| chr20 | 8566792 | 8889612 | 322820 | America |
| chr20 | 16513316 | 16683777 | 170461 | America |
| chr21 | 30031030 | 30864811 | 833781 | Bantu |
| chr21 | 44172913 | 44422295 | 249382 | Bantu |
| chr21 | 46032094 | 46158737 | 126643 | Bantu |
| chr21 | 46982335 | 47322335 | 340000 | Middle East |
| chr21 | 17817942 | 18114357 | 296415 | East Asia |
| chr21 | 29966286 | 31178625 | 1212339 | South Asia |
| chr21 | 40326522 | 41030756 | 704234 | America |
| chr21 | 41049045 | 41278301 | 229256 | America |
| chr21 | 15861455 | 15984177 | 122722 | America |
| chr22 | 31530730 | 32289918 | 759188 | Bantu |
| chr22 | 46447097 | 46863377 | 416280 | Middle East |
| chr22 | 18526789 | 18918824 | 392035 | Middle East |
| chr22 | 35518987 | 35797832 | 278845 | Middle East |
| chr22 | 32275353 | 32699672 | 424319 | East Asia |
| chr22 | 36478027 | 36935795 | 457768 | East Asia |
| chr22 | 46447097 | 46863377 | 416280 | South Asia |
| chr22 | 28244191 | 29198150 | 953959 | South Asia |
| chr22 | 25619339 | 25950749 | 331410 | South Asia |
| chr22 | 35519850 | 35795412 | 275562 | Europe |
| chr22 | 49912406 | 50491712 | 579306 | Europe |
| chr22 | 30654464 | 30921370 | 266906 | Europe |
| chr22 | 49855674 | 50278567 | 422893 | America |
| chrX | 121993027 | 122366636 | 373609 | Bantu |
| chrX | 126640531 | 127637654 | 997123 | Bantu |
| chrX | 109096770 | 110400647 | 1303877 | Bantu |
| chrX | 34986964 | 35753528 | 766564 | Bantu |
| chrX | 65922033 | 67286067 | 1364034 | Bantu |
| chrX | 137952577 | 138233542 | 280965 | Bantu |
| chrX | 34225721 | 36560061 | 2334340 | Middle East |
| chrX | 109689152 | 111504315 | 1815163 | Middle East |
| chrX | 98117578 | 99486285 | 1368707 | Middle East |
| chrX | 97004924 | 97760765 | 755841 | Middle East |
| chrX | 30026227 | 30597320 | 571093 | Middle East |
| chrX | 18859525 | 20417949 | 1558424 | Middle East |
| chrX | 5728176 | 6134767 | 406591 | Middle East |
| chrX | 153189819 | 153627145 | 437326 | Middle East |
| chrX | 67177760 | 68076640 | 898880 | East Asia |
| chrX | 97108437 | 97837882 | 729445 | East Asia |
| chrX | 14035798 | 15163507 | 1127709 | East Asia |
| chrX | 150438543 | 150901389 | 462846 | East Asia |
| chrX | 64405169 | 68053507 | 3648338 | South Asia |
| chrX | 20522619 | 21802998 | 1280379 | South Asia |
| chrX | 108966425 | 111694354 | 2727929 | South Asia |
| chrX | 30272489 | 30546430 | 273941 | South Asia |
| chrX | 121222635 | 121942321 | 719686 | South Asia |
| chrX | 125843757 | 127823996 | 1980239 | Europe |
| chrX | 95072450 | 95872001 | 799551 | Europe |
| chrX | 98224405 | 99486285 | 1261880 | Europe |
| chrX | 65435257 | 67923735 | 2488478 | Europe |
| chrX | 106736820 | 111555208 | 4818388 | America |
| chrX | 5650083 | 6135610 | 485527 | America |
