# Supplementary material for: CpG Islands Undermethylation in Human Genomic Regions under Selective Pressure
Source: PLoS One. 2011 Aug 2;6(8):e23156. doi: 10.1371/journal.pone.0023156 (PMC3149076; doi:10.1371/journal.pone.0023156)
Supplement: Table S4 — Lists, for each cell type, the mean methylation of CGIs inside HIRs (with its standard error), the mean methylation of CGIs localized outside these regions (with its standard error), the number of CGIs inside HIRs, the number of CGIs localized outside HIRs and the Bootstrap p-values. (DOC) [file pone.0023156.s007.doc]

| **Cell ID** | **Cell type** | **HIR CGIs mean** | **HIR SE** | **Other CGIs mean** | **Other SE** | **n. HIR CGIs** | **n. other CGIs** | **Bootstrap p-value** |
| --- | --- | --- | --- | --- | --- | --- | --- | --- |
| Hek293 | cancer | 18.07209546 | 0.74963397 | 21.71357874 | 0.27182048 | 1662 | 15159 | < 1,0E-04 |
| MCF-7 | cancer | 28.3134172 | 0.90845651 | 33.25740612 | 0.31383639 | 1784 | 16415 | < 1,0E-04 |
| Hepg2 | cancer | 23.58733521 | 0.81826007 | 27.39282804 | 0.28077965 | 1764 | 16427 | 1,0E-04 |
| Cmk | cancer | 26.3994764 | 0.89198779 | 33.63190574 | 0.31407055 | 1741 | 16102 | < 1,0E-04 |
| NB4 | cancer | 27.10041988 | 0.86443758 | 31.93859768 | 0.29668278 | 1745 | 16100 | < 1,0E-04 |
| NT2-D1 | cancer | 12.67613946 | 0.70799264 | 16.301809 | 0.26401909 | 1616 | 14855 | < 1,0E-04 |
| Gm19239 | EBV | 13.61689916 | 0.64389488 | 17.88822672 | 0.24354854 | 1635 | 15223 | < 1,0E-04 |
| Gm19240 | EBV | 17.0858443 | 0.67483608 | 20.74296261 | 0.24533451 | 1811 | 16766 | < 1,0E-04 |
| Ag04449 | normal | 8.291692217 | 0.42508266 | 10.14338957 | 0.16246635 | 1696 | 15412 | 1,0E-04 |
| Ag04450 | normal | 11.03531603 | 0.56654663 | 13.94272058 | 0.21097382 | 1758 | 16285 | < 1,0E-04 |
| Ag09309 | normal | 13.68236452 | 0.59444949 | 17.1290823 | 0.22176914 | 1758 | 16355 | < 1,0E-04 |
| Ag09319 | normal | 11.61979758 | 0.59526593 | 14.74437777 | 0.22517808 | 1691 | 15459 | < 1,0E-04 |
| Ag10803 | normal | 12.37297695 | 0.58687882 | 16.00796514 | 0.22029061 | 1855 | 17306 | < 1,0E-04 |
| Fibrobl | normal | 14.11595976 | 0.630049 | 17.49915036 | 0.23169448 | 1701 | 15907 | < 1,0E-04 |
| HAEpiC | normal | 10.42137587 | 0.56003811 | 14.11197435 | 0.21809566 | 1764 | 16270 | < 1,0E-04 |
| HCF | normal | 9.07611084 | 0.55552218 | 12.9804742 | 0.22368603 | 1591 | 14546 | < 1,0E-04 |
| HCM | normal | 10.40447994 | 0.56516963 | 13.60023807 | 0.21422817 | 1817 | 16829 | < 1,0E-04 |
| HEEpiC | normal | 10.04693673 | 0.54926804 | 13.38862614 | 0.21297418 | 1729 | 15915 | < 1,0E-04 |
| HIPEpiC | normal | 10.41191083 | 0.5493907 | 13.52147836 | 0.21222731 | 1742 | 15980 | < 1,0E-04 |
| HMEC | normal | 12.72713219 | 0.61298982 | 16.35859901 | 0.23280776 | 1736 | 16089 | < 1,0E-04 |
| HNPCEpiC | normal | 10.14362759 | 0.54589589 | 13.08930351 | 0.20736638 | 1801 | 16630 | < 1,0E-04 |
| HRCEpiC | normal | 9.108447913 | 0.56073348 | 12.00655231 | 0.21357911 | 1646 | 15161 | < 1,0E-04 |
| HSMMtube | normal | 18.00726159 | 0.66202673 | 21.25326949 | 0.2386281 | 1789 | 16680 | < 1,0E-04 |
| NHBE | normal | 11.02629679 | 0.57314939 | 14.52355975 | 0.22030469 | 1767 | 16389 | < 1,0E-04 |
| Skmc | normal | 10.79080888 | 0.59161788 | 14.86922907 | 0.22582651 | 1741 | 16221 | < 1,0E-04 |
