# Supplementary material for: CpG Islands Undermethylation in Human Genomic Regions under Selective Pressure
Source: PLoS One. 2011 Aug 2;6(8):e23156. doi: 10.1371/journal.pone.0023156 (PMC3149076; doi:10.1371/journal.pone.0023156)
Supplement: Table S5 — Lists, for each 5SLR identified, the chromosome, the start position, the end position and the total length. Genomic coordinates refer to assembly GRCh37/hg19. (DOC) [file pone.0023156.s008.doc]

| **Regions** | **Start** | **End** | **Length** |
| --- | --- | --- | --- |
| chr1 | 29250635 | 29582124 | 331489 |
| chr1 | 46026531 | 46214183 | 187652 |
| chr1 | 63862069 | 64092146 | 230077 |
| chr1 | 114057822 | 114381300 | 323478 |
| chr1 | 115116433 | 115378793 | 262360 |
| chr1 | 46554517 | 46718374 | 163857 |
| chr1 | 51008171 | 51235816 | 227645 |
| chr1 | 78052717 | 78289590 | 236873 |
| chr1 | 94639336 | 94839130 | 199794 |
| chr1 | 97229888 | 97509581 | 279693 |
| chr1 | 98053562 | 98445381 | 391819 |
| chr1 | 151286575 | 151611883 | 325308 |
| chr1 | 199900613 | 200030369 | 129756 |
| chr1 | 210136341 | 210369242 | 232901 |
| chr1 | 243662053 | 243895373 | 233320 |
| chr1 | 85066633 | 85177704 | 111071 |
| chr2 | 63927885 | 64188651 | 260766 |
| chr2 | 74448828 | 74715238 | 266410 |
| chr2 | 99573995 | 99832771 | 258776 |
| chr2 | 103756037 | 103972017 | 215980 |
| chr2 | 144697382 | 145176389 | 479007 |
| chr2 | 145592590 | 145791678 | 199088 |
| chr2 | 148547241 | 149002548 | 455307 |
| chr2 | 176927165 | 177270516 | 343351 |
| chr2 | 205421745 | 205651824 | 230079 |
| chr2 | 15412979 | 15586646 | 173667 |
| chr2 | 22662161 | 22828842 | 166681 |
| chr2 | 31733656 | 31971218 | 237562 |
| chr2 | 32718848 | 32917771 | 198923 |
| chr2 | 37095793 | 37258412 | 162619 |
| chr2 | 43411503 | 43747885 | 336382 |
| chr2 | 57948185 | 58116477 | 168292 |
| chr2 | 61424903 | 61742537 | 317634 |
| chr2 | 62981797 | 63289808 | 308011 |
| chr2 | 72690520 | 72852907 | 162387 |
| chr2 | 73549924 | 73794209 | 244285 |
| chr2 | 99185422 | 99478291 | 292869 |
| chr2 | 123575222 | 123715501 | 140279 |
| chr2 | 135845130 | 136125165 | 280035 |
| chr2 | 142960140 | 143128724 | 168584 |
| chr2 | 146134867 | 146337410 | 202543 |
| chr2 | 152247420 | 152435284 | 187864 |
| chr2 | 155700922 | 155938213 | 237291 |
| chr2 | 187897747 | 188105218 | 207471 |
| chr2 | 232835431 | 233180541 | 345110 |
| chr3 | 49097085 | 49399863 | 302778 |
| chr3 | 94365581 | 94623235 | 257654 |
| chr3 | 95238967 | 95423773 | 184806 |
| chr3 | 99494533 | 99834482 | 339949 |
| chr3 | 136208787 | 136429395 | 220608 |
| chr3 | 13969054 | 14217300 | 248246 |
| chr3 | 25797576 | 25976557 | 178981 |
| chr3 | 44325263 | 44663213 | 337950 |
| chr3 | 48613019 | 48895220 | 282201 |
| chr3 | 58841784 | 59137547 | 295763 |
| chr3 | 71400071 | 71615018 | 214947 |
| chr3 | 79204857 | 79537773 | 332916 |
| chr3 | 110619507 | 110904347 | 284840 |
| chr3 | 114684268 | 114906146 | 221878 |
| chr3 | 119975093 | 120114914 | 139821 |
| chr3 | 130635092 | 130860757 | 225665 |
| chr3 | 155761633 | 156114898 | 353265 |
| chr3 | 156493013 | 156744033 | 251020 |
| chr3 | 159979892 | 160278818 | 298926 |
| chr3 | 176706064 | 176840396 | 134332 |
| chr3 | 180964991 | 181123818 | 158827 |
| chr3 | 181695505 | 181910529 | 215024 |
| chr4 | 128619572 | 128993304 | 373732 |
| chr4 | 34010143 | 34247945 | 237802 |
| chr4 | 46173112 | 46557143 | 384031 |
| chr4 | 67417795 | 67622971 | 205176 |
| chr4 | 74983811 | 75148509 | 164698 |
| chr4 | 81177928 | 81697216 | 519288 |
| chr4 | 98214952 | 98529524 | 314572 |
| chr4 | 123960051 | 124100556 | 140505 |
| chr4 | 172431101 | 172637662 | 206561 |
| chr5 | 109035495 | 109234306 | 198811 |
| chr5 | 19608621 | 19981207 | 372586 |
| chr5 | 43889881 | 44105084 | 215203 |
| chr5 | 45050586 | 45393754 | 343168 |
| chr5 | 61682074 | 61960620 | 278546 |
| chr5 | 93050452 | 93551028 | 500576 |
| chr5 | 114116640 | 114350704 | 234064 |
| chr5 | 131013414 | 131263355 | 249941 |
| chr5 | 17921280 | 18056522 | 135242 |
| chr5 | 27016109 | 27151405 | 135296 |
| chr5 | 37380577 | 37649448 | 268871 |
| chr5 | 71456746 | 71647477 | 190731 |
| chr5 | 81284883 | 81637960 | 353077 |
| chr5 | 86549391 | 86844673 | 295282 |
| chr5 | 126999041 | 127242725 | 243684 |
| chr5 | 160787118 | 161026677 | 239559 |
| chr6 | 79526516 | 79820785 | 294269 |
| chr6 | 126668173 | 126989092 | 320919 |
| chr6 | 132102752 | 132196457 | 93705 |
| chr6 | 140338210 | 140976317 | 638107 |
| chr6 | 45332304 | 45597525 | 265221 |
| chr6 | 49788953 | 50101371 | 312418 |
| chr6 | 84298676 | 84482187 | 183511 |
| chr6 | 90706068 | 90847204 | 141136 |
| chr6 | 98985278 | 99143313 | 158035 |
| chr6 | 128110771 | 128305888 | 195117 |
| chr7 | 69025009 | 69636926 | 611917 |
| chr7 | 98521518 | 98713281 | 191763 |
| chr7 | 132988393 | 133189530 | 201137 |
| chr7 | 18379007 | 18586307 | 207300 |
| chr7 | 40050109 | 40192009 | 141900 |
| chr7 | 41571216 | 41871572 | 300356 |
| chr7 | 43605654 | 43888375 | 282721 |
| chr7 | 48654708 | 48827838 | 173130 |
| chr7 | 93759190 | 93985638 | 226448 |
| chr7 | 107010512 | 107177005 | 166493 |
| chr7 | 114411347 | 114629622 | 218275 |
| chr7 | 121976180 | 122495427 | 519247 |
| chr7 | 127178538 | 127662335 | 483797 |
| chr8 | 34598254 | 34831202 | 232948 |
| chr8 | 49252900 | 49477598 | 224698 |
| chr8 | 53439456 | 53638576 | 199120 |
| chr8 | 64868710 | 65123925 | 255215 |
| chr8 | 116359583 | 116569557 | 209974 |
| chr8 | 19385418 | 19497670 | 112252 |
| chr8 | 28773614 | 29154132 | 380518 |
| chr8 | 35772643 | 36067503 | 294860 |
| chr8 | 47708415 | 48104073 | 395658 |
| chr8 | 49594702 | 49828725 | 234023 |
| chr8 | 58427937 | 58603646 | 175709 |
| chr8 | 63732189 | 63873882 | 141693 |
| chr8 | 66307235 | 66408755 | 101520 |
| chr8 | 71004379 | 71195443 | 191064 |
| chr8 | 92653592 | 92906609 | 253017 |
| chr8 | 99633644 | 100014611 | 380967 |
| chr8 | 100117902 | 100308266 | 190364 |
| chr8 | 127090898 | 127173121 | 82223 |
| chr9 | 125718241 | 126026082 | 307841 |
| chr9 | 84794785 | 85035221 | 240436 |
| chr9 | 37785698 | 37927533 | 141835 |
| chr9 | 102334727 | 102629879 | 295152 |
| chr10 | 62673306 | 62985661 | 312355 |
| chr10 | 74413470 | 74667147 | 253677 |
| chr10 | 103706040 | 104007262 | 301222 |
| chr10 | 9610081 | 9746948 | 136867 |
| chr10 | 32879965 | 33141717 | 261752 |
| chr10 | 50674530 | 50815585 | 141055 |
| chr10 | 60345768 | 60592816 | 247048 |
| chr10 | 75275890 | 75476096 | 200206 |
| chr10 | 83346626 | 83724563 | 377937 |
| chr10 | 106713807 | 106848420 | 134613 |
| chr10 | 24907707 | 25031090 | 123383 |
| chr11 | 31061804 | 31670889 | 609085 |
| chr11 | 27485795 | 27721022 | 235227 |
| chr11 | 30644423 | 31036216 | 391793 |
| chr11 | 41655528 | 41784303 | 128775 |
| chr11 | 45325027 | 45512886 | 187859 |
| chr11 | 45746288 | 45911305 | 165017 |
| chr11 | 55023837 | 55441682 | 417845 |
| chr11 | 55647914 | 55896015 | 248101 |
| chr11 | 56627239 | 56896648 | 269409 |
| chr11 | 57008535 | 57100848 | 92313 |
| chr11 | 59857388 | 60041190 | 183802 |
| chr11 | 71895114 | 72237309 | 342195 |
| chr11 | 72517367 | 72762960 | 245593 |
| chr11 | 95893439 | 96227949 | 334510 |
| chr11 | 108543742 | 108800455 | 256713 |
| chr12 | 89009747 | 89366763 | 357016 |
| chr12 | 15405896 | 15572168 | 166272 |
| chr12 | 16884746 | 17099885 | 215139 |
| chr12 | 65795003 | 65925191 | 130188 |
| chr12 | 87278562 | 87505777 | 227215 |
| chr12 | 90247389 | 90498984 | 251595 |
| chr13 | 20534301 | 20657647 | 123346 |
| chr13 | 51944276 | 52082437 | 138161 |
| chr13 | 60540445 | 60789907 | 249462 |
| chr13 | 68623480 | 68924857 | 301377 |
| chr13 | 84132000 | 84421285 | 289285 |
| chr14 | 75426604 | 75646308 | 219704 |
| chr14 | 59676151 | 59822731 | 146580 |
| chr14 | 49861650 | 50025588 | 163938 |
| chr14 | 68276249 | 68463491 | 187242 |
| chr14 | 71750112 | 72211600 | 461488 |
| chr14 | 83608854 | 83776600 | 167746 |
| chr14 | 101178423 | 101347971 | 169548 |
| chr14 | 105636111 | 105757770 | 121659 |
| chr15 | 72142918 | 72396120 | 253202 |
| chr15 | 49675957 | 49862449 | 186492 |
| chr15 | 64593136 | 64952886 | 359750 |
| chr15 | 84016770 | 84224384 | 207614 |
| chr15 | 84319667 | 84496111 | 176444 |
| chr16 | 34269791 | 34737702 | 467911 |
| chr16 | 61845083 | 62098061 | 252978 |
| chr16 | 63811605 | 64117746 | 306141 |
| chr16 | 34917709 | 35149023 | 231314 |
| chr16 | 46805547 | 47144825 | 339278 |
| chr17 | 28223981 | 28512879 | 288898 |
| chr17 | 27410713 | 27594951 | 184238 |
| chr17 | 30947344 | 31213911 | 266567 |
| chr17 | 33121487 | 33398915 | 277428 |
| chr17 | 58271331 | 58508582 | 237251 |
| chr17 | 62469023 | 62706874 | 237851 |
| chr17 | 67929661 | 68017584 | 87923 |
| chr18 | 34395780 | 34824354 | 428574 |
| chr18 | 31557831 | 31867235 | 309404 |
| chr18 | 41300552 | 41565496 | 264944 |
| chr19 | 10931683 | 11147333 | 215650 |
| chr20 | 33474216 | 33644358 | 170142 |
| chr20 | 11423194 | 11555408 | 132214 |
| chr20 | 13388946 | 13660066 | 271120 |
| chr20 | 30247858 | 30480632 | 232774 |
| chr20 | 34425967 | 34610890 | 184923 |
| chr20 | 32988114 | 33235384 | 247270 |
| chr21 | 38658252 | 38867218 | 208966 |
| chr22 | 28575508 | 28914746 | 339238 |
| chrX | 63515628 | 63803599 | 287971 |
