# Supplementary material for: CpG Islands Undermethylation in Human Genomic Regions under Selective Pressure
Source: PLoS One. 2011 Aug 2;6(8):e23156. doi: 10.1371/journal.pone.0023156 (PMC3149076; doi:10.1371/journal.pone.0023156)
Supplement: Table S6 — Lists, for each cell type, the mean methylation of CGIs inside 5SLRs (with its standard error), the mean methylation of CGIs localized outside these regions (with its standard error), the number of CGIs inside 5SLRs s, the number of CGIs localized outside 5SLRs and the Bootstrap p-values. (DOC) [file pone.0023156.s009.doc]

| **Cell ID** | **Cell type** | **5SLR CGIs mean** | **5SLR SE** | **Other CGIs mean** | **Other SE** | **n. 5SLR CGIs** | **n. other CGIs** | **Bootstrap p-value** |
| --- | --- | --- | --- | --- | --- | --- | --- | --- |
| Hek293 | cancer | 14.13274539 | 1.8511369 | 21.45874374 | 0.25828709 | 241 | 16580 | 1.0E-04 |
| MCF-7 | cancer | 27.71896703 | 2.41106012 | 32.84600737 | 0.29918481 | 260 | 17939 | 0.0177 |
| Hepg2 | cancer | 22.04356073 | 2.04318492 | 27.0943291 | 0.26797811 | 254 | 17937 | 0.0115 |
| Cmk | cancer | 25.47229476 | 2.31296117 | 33.0334249 | 0.29927342 | 253 | 17590 | 0.0014 |
| NB4 | cancer | 25.0108622 | 2.26837969 | 31.55868889 | 0.28301359 | 254 | 17591 | 0.0022 |
| NT2-D1 | cancer | 11.17169623 | 1.67069663 | 16.01758022 | 0.25061011 | 243 | 16228 | 0.0069 |
| Gm19239 | EBV | 10.98331376 | 1.41763614 | 17.56968601 | 0.2311772 | 245 | 16613 | 1.0E-04 |
| Gm19240 | EBV | 11.81273641 | 1.36922079 | 20.50909299 | 0.23348113 | 262 | 18315 | <1.0E-04 |
| Ag04449 | normal | 7.649343629 | 0.98891071 | 9.995338002 | 0.15394158 | 259 | 16849 | 0.0256 |
| Ag04450 | normal | 7.43814311 | 1.07195526 | 13.75182071 | 0.20059277 | 264 | 17779 | <1.0E-04 |
| Ag09309 | normal | 12.56376747 | 1.470223 | 16.85736998 | 0.21045972 | 265 | 17848 | 0.0043 |
| Ag09319 | normal | 8.559445713 | 1.25525234 | 14.52322794 | 0.21365955 | 250 | 16900 | 1.0E-04 |
| Ag10803 | normal | 9.817678077 | 1.30871342 | 15.74044319 | 0.20913789 | 273 | 18888 | 2.0E-04 |
| Fibrobl | normal | 11.861235 | 1.46617254 | 17.24912499 | 0.22019253 | 251 | 17357 | 0.0013 |
| HAEpiC | normal | 7.342581647 | 1.13871897 | 13.84691597 | 0.20668094 | 266 | 17768 | 1.0E-04 |
| HCF | normal | 7.011662842 | 1.220579 | 12.67769232 | 0.21138466 | 234 | 15903 | 3.0E-04 |
| HCM | normal | 6.637513291 | 1.11430818 | 13.38838552 | 0.20339969 | 275 | 18371 | <1.0E-04 |
| HEEpiC | normal | 8.023852996 | 1.22452669 | 13.13591306 | 0.20173261 | 258 | 17386 | 3.0E-04 |
| HIPEpiC | normal | 7.311095836 | 1.14318102 | 13.3044253 | 0.20114004 | 262 | 17460 | <1.0E-04 |
| HMEC | normal | 9.655115391 | 1.28904814 | 16.09928353 | 0.22093885 | 261 | 17564 | <1.0E-04 |
| HNPCEpiC | normal | 6.66867062 | 1.03489542 | 12.89298353 | 0.19688426 | 271 | 18160 | <1.0E-04 |
| HRCEpiC | normal | 6.161202927 | 1.0698717 | 11.80499639 | 0.20272315 | 245 | 16562 | <1.0E-04 |
| HSMMtube | normal | 13.95838672 | 1.38350565 | 21.03812762 | 0.22722535 | 259 | 18210 | <1.0E-04 |
| NHBE | normal | 8.672499068 | 1.25910838 | 14.26513149 | 0.20885555 | 266 | 17890 | 2.0E-04 |
| Skmc | normal | 7.906367112 | 1.17897318 | 14.57038231 | 0.21436746 | 260 | 17702 | <1.0E-04 |
