# Supplementary material for: CpG Islands Undermethylation in Human Genomic Regions under Selective Pressure
Source: PLoS One. 2011 Aug 2;6(8):e23156. doi: 10.1371/journal.pone.0023156 (PMC3149076; doi:10.1371/journal.pone.0023156)
Supplement: Table S7 — Lists, for each cell type, the mean methylation of CGIs containing CEs (with its standard error), the mean methylation of CGIs that not contain CEs (with its standard error), the number of CE CGIs, the number of non-CE CGIs and the Bootstrap p-values. (DOC) [file pone.0023156.s010.doc]

| **Cell ID** | **Cell type** | **CE CGIs mean** | **CE SE** | **Other CGIs mean** | **Other SE** | **n. CE CGIs** | **n. other CGIs** | **Bootstrap p-value** |
| --- | --- | --- | --- | --- | --- | --- | --- | --- |
| Hek293 | cancer | 18.06194902 | 0.30389391 | 26.63876414 | 0.4472349 | 10365 | 6456 | <1.0E-04 |
| MCF-7 | cancer | 29.31174071 | 0.3666015 | 38.08065182 | 0.4934211 | 11016 | 7183 | <1.0E-04 |
| Hepg2 | cancer | 24.10220364 | 0.32920185 | 31.47347397 | 0.44026143 | 10981 | 7210 | <1.0E-04 |
| Cmk | cancer | 30.52155783 | 0.37046424 | 36.63095243 | 0.49000397 | 10820 | 7023 | <1.0E-04 |
| NB4 | cancer | 28.67202877 | 0.3463578 | 35.77711741 | 0.46939085 | 10829 | 7016 | <1.0E-04 |
| NT2-D1 | cancer | 12.66381809 | 0.28249896 | 21.215268 | 0.45310448 | 10149 | 6322 | <1.0E-04 |
| Gm19239 | EBV | 14.64260305 | 0.2693243 | 21.94428629 | 0.40303282 | 10321 | 6537 | <1.0E-04 |
| Gm19240 | EBV | 17.38779645 | 0.27665389 | 24.94318509 | 0.39712404 | 11204 | 7373 | <1.0E-04 |
| Ag04449 | normal | 8.162944221 | 0.17489162 | 12.76902457 | 0.27540567 | 10434 | 6674 | <1.0E-04 |
| Ag04450 | normal | 11.42000275 | 0.23610187 | 17.08627404 | 0.34446075 | 10912 | 7131 | <1.0E-04 |
| Ag09309 | normal | 14.45463991 | 0.25464626 | 20.3435999 | 0.35121572 | 10916 | 7197 | <1.0E-04 |
| Ag09319 | normal | 11.9928598 | 0.24871994 | 18.27074267 | 0.37295181 | 10475 | 6675 | <1.0E-04 |
| Ag10803 | normal | 13.05760133 | 0.25026711 | 19.48613818 | 0.35094952 | 11416 | 7745 | <1.0E-04 |
| Fibrobl | normal | 14.65374098 | 0.26273602 | 21.06414299 | 0.37384171 | 10690 | 6918 | <1.0E-04 |
| HAEpiC | normal | 11.11014802 | 0.23744364 | 17.78682268 | 0.36280708 | 10901 | 7133 | <1.0E-04 |
| HCF | normal | 10.25670137 | 0.24188447 | 16.36772469 | 0.3777372 | 9961 | 6176 | <1.0E-04 |
| HCM | normal | 11.02712334 | 0.2386924 | 16.71753014 | 0.35013204 | 11235 | 7411 | <1.0E-04 |
| HEEpiC | normal | 10.74731068 | 0.23342271 | 16.64781495 | 0.35385824 | 10725 | 6919 | <1.0E-04 |
| HIPEpiC | normal | 10.83446026 | 0.2315216 | 16.90413784 | 0.35407515 | 10769 | 6953 | <1.0E-04 |
| HMEC | normal | 13.37336375 | 0.25791805 | 20.05535694 | 0.38294662 | 10805 | 7020 | <1.0E-04 |
| HNPCEpiC | normal | 10.51655713 | 0.22922112 | 16.27207527 | 0.34130963 | 11114 | 7317 | <1.0E-04 |
| HRCEpiC | normal | 9.776230222 | 0.23552555 | 14.82870027 | 0.35643076 | 10332 | 6475 | <1.0E-04 |
| HSMMtube | normal | 17.72691312 | 0.26767559 | 25.78575942 | 0.38746289 | 11108 | 7361 | <1.0E-04 |
| NHBE | normal | 11.61694328 | 0.24213548 | 18.12160123 | 0.36439618 | 10993 | 7163 | <1.0E-04 |
| Skmc | normal | 11.82268113 | 0.25106864 | 18.53088857 | 0.36932762 | 10863 | 7099 | <1.0E-04 |
