# Supplementary material for: CpG Islands Undermethylation in Human Genomic Regions under Selective Pressure
Source: PLoS One. 2011 Aug 2;6(8):e23156. doi: 10.1371/journal.pone.0023156 (PMC3149076; doi:10.1371/journal.pone.0023156)
Supplement: Table S8 — Lists, for each cell type, the mean methylation of HIR+CE CGIs (with its standard error), the mean methylation of CE CGIs (with its standard error), the number of HIR+CE CGIs, the number of CE CGIs and the Bootstrap p-values. (DOC) [file pone.0023156.s011.doc]

| **Cell ID** | **Cell type** | **HIR+CE CGIs mean** | **HIR+CE SE** | **CE CGIs mean** | **CE SE** | **n. HIR+CE CGIs** | **n. CE CGIs** | **Bootstrap p-value** |
| --- | --- | --- | --- | --- | --- | --- | --- | --- |
| Hek293 | cancer | 15.83623485 | 0.89925163 | 18.30541256 | 0.32238172 | 1022 | 9343 | 0.0069 |
| MCF-7 | cancer | 24.78486437 | 1.10807098 | 29.80379249 | 0.38789151 | 1080 | 9936 | < 1.E-04 |
| Hepg2 | cancer | 20.94443157 | 1.00181754 | 24.44453201 | 0.34819505 | 1074 | 9907 | 0.0011 |
| Cmk | cancer | 24.43245785 | 1.10632827 | 31.18149102 | 0.39215476 | 1058 | 9762 | < 1.E-04 |
| NB4 | cancer | 24.48509553 | 1.05010054 | 29.12728863 | 0.36637262 | 1062 | 9767 | < 1.E-04 |
| NT2-D1 | cancer | 9.818266859 | 0.78875638 | 12.97139486 | 0.30103731 | 990 | 9159 | 7.E-04 |
| Gm19239 | EBV | 11.78969143 | 0.764194 | 14.9486766 | 0.28655855 | 1000 | 9321 | 1.E-04 |
| Gm19240 | EBV | 14.98866755 | 0.80971263 | 17.64898427 | 0.29372972 | 1100 | 10104 | 1.E-03 |
| Ag04449 | normal | 7.017109602 | 0.48293697 | 8.28858024 | 0.18666602 | 1031 | 9403 | 0.013 |
| Ag04450 | normal | 9.514735893 | 0.68227508 | 11.62520914 | 0.25091804 | 1061 | 9851 | 0.0025 |
| Ag09309 | normal | 12.12900686 | 0.73520006 | 14.70528151 | 0.27062392 | 1062 | 9854 | 9.E-04 |
| Ag09319 | normal | 9.680989007 | 0.69333385 | 12.24551792 | 0.26517444 | 1032 | 9443 | 7.E-04 |
| Ag10803 | normal | 10.67151203 | 0.70994591 | 13.31510631 | 0.26636641 | 1112 | 10304 | 7.E-04 |
| Fibrobl | normal | 12.49566357 | 0.76714702 | 14.88582598 | 0.27896015 | 1038 | 9652 | 0.0039 |
| HAEpiC | normal | 8.676708592 | 0.65495135 | 11.37417892 | 0.25329867 | 1067 | 9834 | 2.E-04 |
| HCF | normal | 7.853319139 | 0.6583744 | 10.51984736 | 0.25836529 | 983 | 8978 | < 1.E-04 |
| HCM | normal | 8.903958885 | 0.6712296 | 11.25663199 | 0.25425245 | 1096 | 10139 | 0.0017 |
| HEEpiC | normal | 8.343485959 | 0.64060805 | 11.00764015 | 0.24909169 | 1048 | 9677 | 2.E-04 |
| HIPEpiC | normal | 8.803586043 | 0.63911698 | 11.05664923 | 0.24705585 | 1062 | 9707 | 0.0015 |
| HMEC | normal | 10.33919878 | 0.70639778 | 13.69995249 | 0.27518135 | 1050 | 9755 | < 1.E-04 |
| HNPCEpiC | normal | 8.630212897 | 0.64394071 | 10.72105062 | 0.24421423 | 1087 | 10027 | 0.0028 |
| HRCEpiC | normal | 7.883028293 | 0.66014666 | 9.982026289 | 0.25099236 | 1013 | 9319 | 0.0027 |
| HSMMtube | normal | 15.67666393 | 0.7918037 | 17.94726892 | 0.28389121 | 1078 | 10030 | 0.0042 |
| NHBE | normal | 9.133926979 | 0.67076771 | 11.88441048 | 0.25816884 | 1069 | 9924 | 2.E-04 |
| Skmc | normal | 9.147031134 | 0.69973955 | 12.11139481 | 0.26756361 | 1058 | 9805 | < 1.E-04 |
