# Supplementary material for: CpG Islands Undermethylation in Human Genomic Regions under Selective Pressure
Source: PLoS One. 2011 Aug 2;6(8):e23156. doi: 10.1371/journal.pone.0023156 (PMC3149076; doi:10.1371/journal.pone.0023156)
Supplement: Table S9 — Lists, for each cell type, the mean methylation of 5SLR+CE CGIs (with its standard error), the mean methylation of CE CGIs (with its standard error), the number of 5SLR+CE CGIs, the number of CE CGIs and the Bootstrap p-values. (DOC) [file pone.0023156.s012.doc]

| **Cell ID** | **Cell type** | **5LSR+CE CGIs mean** | **5LSR +CE SE** | **CE CGIs mean** | **CE SE** | **n. 5LSR +CE CGIs** | **n. CE CGIs** | **Bootstrap p-value** |
| --- | --- | --- | --- | --- | --- | --- | --- | --- |
| Hek293 | cancer | 10.37628825 | 1.90918117 | 18.17939079 | 0.30701808 | 156 | 10209 | 1.0E-04 |
| MCF-7 | cancer | 22.93389404 | 2.80151076 | 29.40812542 | 0.36966261 | 164 | 10852 | 0.0154 |
| Hepg2 | cancer | 18.90791051 | 2.32056151 | 24.1799812 | 0.3322738 | 162 | 10819 | 0.0242 |
| Cmk | cancer | 23.01581493 | 2.73381193 | 30.63492912 | 0.37369178 | 161 | 10659 | 0.0057 |
| NB4 | cancer | 22.02005809 | 2.66749511 | 28.77368555 | 0.34920252 | 163 | 10666 | 0.0066 |
| NT2-D1 | cancer | 5.441370892 | 1.1917729 | 12.77730129 | 0.28618458 | 157 | 9992 | 1.0E-04 |
| Gm19239 | EBV | 7.712200781 | 1.33052292 | 14.74827021 | 0.27254793 | 155 | 10166 | 2.0E-04 |
| Gm19240 | EBV | 8.662255749 | 1.29448932 | 17.51982193 | 0.27997111 | 167 | 11037 | <1.0E-04 |
| Ag04449 | normal | 5.637730061 | 0.92448072 | 8.20301918 | 0.17703586 | 163 | 10271 | 0.0263 |
| Ag04450 | normal | 4.613737254 | 0.83680997 | 11.52707329 | 0.23931281 | 169 | 10743 | <1.0E-04 |
| Ag09309 | normal | 8.804471169 | 1.51644723 | 14.54188903 | 0.25743204 | 166 | 10750 | 0.0013 |
| Ag09319 | normal | 4.964918459 | 0.99712684 | 12.09979784 | 0.25190454 | 157 | 10318 | <1.0E-04 |
| Ag10803 | normal | 7.41177632 | 1.36409965 | 13.14396579 | 0.25315534 | 172 | 11244 | 0.0017 |
| Fibrobl | normal | 9.453901503 | 1.66012629 | 14.73224962 | 0.26545585 | 159 | 10531 | 0.0053 |
| HAEpiC | normal | 5.190239125 | 1.12907275 | 11.20281034 | 0.24040786 | 168 | 10733 | 3.0E-04 |
| HCF | normal | 4.781792145 | 1.15844024 | 10.34154041 | 0.24488216 | 152 | 9809 | 3.0E-04 |
| HCM | normal | 4.342251171 | 1.08079501 | 11.13289663 | 0.24173463 | 175 | 11060 | <1.0E-04 |
| HEEpiC | normal | 5.087948543 | 1.14169091 | 10.83410578 | 0.23625912 | 162 | 10563 | 1.0E-04 |
| HIPEpiC | normal | 4.471057319 | 1.01658838 | 10.93408535 | 0.23448096 | 166 | 10603 | <1.0E-04 |
| HMEC | normal | 5.524053762 | 0.86312715 | 13.49433798 | 0.26138397 | 164 | 10641 | <1.0E-04 |
| HNPCEpiC | normal | 3.873851058 | 0.81003083 | 10.62159215 | 0.23235585 | 173 | 10941 | <1.0E-04 |
| HRCEpiC | normal | 3.569559848 | 0.84418476 | 9.872618458 | 0.23870101 | 158 | 10174 | 1.0E-04 |
| HSMMtube | normal | 10.82291589 | 1.39448125 | 17.8297319 | 0.27075048 | 163 | 10945 | 2.0E-04 |
| NHBE | normal | 5.488231834 | 1.1643759 | 11.71263362 | 0.24513501 | 169 | 10824 | 2.0E-04 |
| Skmc | normal | 5.756426782 | 1.16640023 | 11.91624366 | 0.25420349 | 165 | 10698 | 2.0E-04 |
