# Supplementary material for: CpG Islands Undermethylation in Human Genomic Regions under Selective Pressure
Source: PLoS One. 2011 Aug 2;6(8):e23156. doi: 10.1371/journal.pone.0023156 (PMC3149076; doi:10.1371/journal.pone.0023156)
Supplement: Table S10 — Lists, for each cell type, the number, the mean methylation and the standard error of 5′ CGIs, intragenic CGIs, 3′ CGIs and intergenic CGIs. (DOC) [file pone.0023156.s013.doc]

|  |  | **5’ CGIs** | | | **Intragenic CGIs** | | | **3’ CGIs** | | | **Intergenic CGIs** | | |
| --- | --- | --- | --- | --- | --- | --- | --- | --- | --- | --- | --- | --- | --- |
| **Cell ID** | **Cell type** | **number** | **mean** | **SE** | **number** | **mean** | **SE** | **number** | **mean** | **SE** | **number** | **mean** | **SE** |
| Hek293 | cancer | 10947 | 12.55 | 0.24 | 995 | 64.95 | 1.2 | 746 | 55.61 | 1.46 | 3187 | 31.54 | 0.64 |
| MCF-7 | cancer | 11431 | 21.50 | 0.32 | 1238 | 74.17 | 1 | 888 | 65.73 | 1.31 | 3580 | 47.31 | 0.69 |
| Hepg2 | cancer | 11310 | 17.76 | 0.28 | 1291 | 64.87 | 1 | 966 | 60.73 | 1.26 | 3592 | 34.67 | 0.60 |
| Cmk | cancer | 11386 | 23.22 | 0.33 | 1165 | 70.83 | 1.1 | 843 | 61.89 | 1.38 | 3430 | 46.84 | 0.69 |
| NB4 | cancer | 11343 | 21.75 | 0.31 | 1145 | 70.30 | 1 | 856 | 60.96 | 1.33 | 3473 | 44.21 | 0.65 |
| NT2-D1 | cancer | 10744 | 8.73 | 0.22 | 926 | 57.94 | 1.4 | 723 | 44.37 | 1.63 | 3112 | 22.58 | 0.65 |
| Gm19239 | EBV | 10683 | 9.92 | 0.21 | 1110 | 57.05 | 1.2 | 821 | 45.17 | 1.38 | 3271 | 22.63 | 0.54 |
| Gm19240 | EBV | 11578 | 12.22 | 0.22 | 1314 | 60.63 | 1.1 | 945 | 48.41 | 1.31 | 3683 | 25.71 | 0.54 |
| Ag04449 | normal | 11105 | 5.92 | 0.13 | 880 | 35.20 | 1.2 | 735 | 26.95 | 1.16 | 3377 | 13.33 | 0.37 |
| Ag04450 | normal | 11411 | 7.75 | 0.18 | 1164 | 47.92 | 1.1 | 865 | 35.21 | 1.27 | 3554 | 17.01 | 0.47 |
| Ag09309 | normal | 11365 | 10.04 | 0.2 | 1219 | 53.42 | 1.1 | 874 | 41.84 | 1.28 | 3613 | 20.44 | 0.46 |
| Ag09319 | normal | 10969 | 8.16 | 0.19 | 1043 | 49.83 | 1.2 | 800 | 37.98 | 1.35 | 3348 | 19.19 | 0.51 |
| Ag10803 | normal | 11886 | 8.66 | 0.19 | 1333 | 52.14 | 1 | 969 | 40.00 | 1.24 | 3868 | 19.41 | 0.47 |
| Fibrobl | normal | 11036 | 9.88 | 0.2 | 1218 | 55.32 | 1.1 | 888 | 43.14 | 1.28 | 3454 | 21.27 | 0.50 |
| HAEpiC | normal | 11356 | 7.39 | 0.18 | 1174 | 48.80 | 1.2 | 872 | 35.81 | 1.31 | 3592 | 17.81 | 0.49 |
| HCF | normal | 10325 | 6.61 | 0.18 | 974 | 47.72 | 1.3 | 774 | 35.08 | 1.36 | 3140 | 16.39 | 0.51 |
| HCM | normal | 11704 | 7.09 | 0.18 | 1218 | 48.23 | 1.2 | 918 | 35.37 | 1.27 | 3736 | 16.73 | 0.48 |
| HEEpiC | normal | 11181 | 7.12 | 0.18 | 1143 | 47.11 | 1.2 | 840 | 33.88 | 1.28 | 3462 | 16.74 | 0.49 |
| HIPEpiC | normal | 11280 | 7.46 | 0.18 | 1105 | 47.52 | 1.2 | 840 | 34.57 | 1.3 | 3480 | 16.71 | 0.48 |
| HMEC | normal | 11259 | 9.10 | 0.2 | 1156 | 52.98 | 1.2 | 867 | 40.72 | 1.34 | 3514 | 20.88 | 0.52 |
| HNPCEpiC | normal | 11625 | 6.95 | 0.17 | 1174 | 46.57 | 1.2 | 878 | 33.32 | 1.28 | 3676 | 16.12 | 0.46 |
| HRCEpiC | normal | 10767 | 6.27 | 0.18 | 1026 | 45.96 | 1.3 | 780 | 33.33 | 1.36 | 3260 | 14.78 | 0.48 |
| HSMMtube | normal | 11506 | 12.90 | 0.21 | 1307 | 58.57 | 1 | 938 | 47.63 | 1.23 | 3658 | 26.84 | 0.53 |
| NHBE | normal | 11450 | 7.68 | 0.19 | 1182 | 49.57 | 1.2 | 890 | 37.16 | 1.28 | 3587 | 18.47 | 0.50 |
| Skmc | normal | 11216 | 7.44 | 0.19 | 1238 | 51.56 | 1.1 | 915 | 39.05 | 1.26 | 3572 | 18.44 | 0.50 |

For each cell line, Kruskal Wallis Test, p-value ≤ 2.2 10^-16^
