# Supplementary material for: CpG Islands Undermethylation in Human Genomic Regions under Selective Pressure
Source: PLoS One. 2011 Aug 2;6(8):e23156. doi: 10.1371/journal.pone.0023156 (PMC3149076; doi:10.1371/journal.pone.0023156)
Supplement: Table S11 — Lists, for each cell type and for each CGIs class (5′ CGIs, intragenic CGIs, 3′ CGIs and intergenic CGIs) the number and the mean methylation of CGIs containing CEs (with its standard error), the number and the mean methylation of CGIs that do not contain CEs (with its standard error), and the Bootstrap p-values. (DOC) [file pone.0023156.s014.doc]

|  |  | **5 ' CGIs** | | | | | | | **Intragenic CGIs** | | | | | | | **3' CGIs** | | | | | | | **Intergenic CGIs** | | | | | | |
| --- | --- | --- | --- | --- | --- | --- | --- | --- | --- | --- | --- | --- | --- | --- | --- | --- | --- | --- | --- | --- | --- | --- | --- | --- | --- | --- | --- | --- | --- |
|  |  | **CE CGIs** | | | **Other CGIs** | | |  | **CE CGIs** | | | **Other CGIs** | | |  | **CE CGIs** | | | **Other CGIs** | | |  | **CE CGIs** | | | **Other CGIs** | | |  |
| **Cell ID** | **Cell type** | **n.** | **mean** | **SE** | **n.** | **mean** | **SE** | **p-value** | **n.** | **mean** | **SE** | **n.** | **mean** | **SE** | **p-value** | **n.** | **mean** | **SE** | **n.** | **mean** | **SE** | **p-value** | **n.** | **mean** | **SE** | **n.** | **mean** | **SE** | **p-value** |
| Hek293 | cancer | 7117 | 11.31 | 0.28 | 3831 | 14.85 | 0.45 | < 10^-4^ | 526 | 64.80 | 1.65 | 469 | 65.11 | 1.72 | 0.4456 | 445 | 57.70 | 1.87 | 301 | 52.53 | 2.34 | 0.9589 | 1228 | 23.56 | 0.94 | 1960 | 36.52 | 0.84 | < 10^-4^ |
| MCF-7 | cancer | 7376 | 20.58 | 0.38 | 4056 | 23.18 | 0.56 | < 10^-4^ | 646 | 74.77 | 1.36 | 592 | 73.52 | 1.44 | 0.7359 | 518 | 67.43 | 1.70 | 370 | 63.34 | 2.05 | 0.9417 | 1306 | 40.35 | 1.11 | 2275 | 51.30 | 0.87 | < 10^-4^ |
| Hepg2 | cancer | 7302 | 16.61 | 0.34 | 4009 | 19.84 | 0.52 | < 10^-4^ | 680 | 66.66 | 1.40 | 611 | 62.89 | 1.50 | 0.9700 | 559 | 62.73 | 1.64 | 407 | 57.98 | 1.97 | 0.9692 | 1300 | 27.76 | 0.94 | 2293 | 38.57 | 0.75 | < 10^-4^ |
| Cmk | cancer | 7338 | 22.94 | 0.40 | 4049 | 23.71 | 0.57 | 0.1354 | 617 | 73.28 | 1.41 | 548 | 68.08 | 1.58 | 0.9924 | 488 | 64.18 | 1.77 | 355 | 58.73 | 2.17 | 0.974 | 1261 | 41.65 | 1.11 | 2170 | 49.84 | 0.88 | < 10^-4^ |
| NB4 | cancer | 7325 | 21.23 | 0.37 | 4019 | 22.70 | 0.53 | 0.0100 | 605 | 70.85 | 1.40 | 540 | 69.68 | 1.51 | 0.7146 | 496 | 63.82 | 1.67 | 360 | 57.01 | 2.15 | 0.9952 | 1274 | 37.67 | 1.01 | 2200 | 47.99 | 0.83 | < 10^-4^ |
| NT2-D1 | cancer | 6995 | 7.71 | 0.26 | 3750 | 10.61 | 0.42 | < 10^-4^ | 484 | 55.41 | 1.99 | 442 | 60.71 | 2.02 | 0.0332 | 425 | 45.31 | 2.12 | 298 | 43.04 | 2.54 | 0.7574 | 1198 | 13.15 | 0.83 | 1915 | 28.47 | 0.90 | < 10^-4^ |
| Gm19239 | EBV | 6964 | 8.99 | 0.24 | 3720 | 11.65 | 0.40 | < 10^-4^ | 587 | 56.50 | 1.61 | 523 | 57.67 | 1.66 | 0.3053 | 484 | 45.70 | 1.80 | 337 | 44.41 | 2.17 | 0.6844 | 1225 | 14.73 | 0.72 | 2047 | 27.35 | 0.73 | < 10^-4^ |
| Gm19240 | EBV | 7469 | 11.11 | 0.26 | 4110 | 14.23 | 0.41 | < 10^-4^ | 698 | 60.39 | 1.47 | 616 | 60.90 | 1.51 | 0.4053 | 547 | 49.79 | 1.73 | 398 | 46.52 | 2.00 | 0.8881 | 1334 | 17.31 | 0.74 | 2350 | 30.46 | 0.71 | < 10^-4^ |
| Ag04449 | normal | 7193 | 5.37 | 0.15 | 3913 | 6.93 | 0.25 | < 10^-4^ | 448 | 32.50 | 1.63 | 432 | 38.00 | 1.64 | 0.0103 | 425 | 28.03 | 1.56 | 310 | 25.48 | 1.74 | 0.8632 | 1274 | 8.83 | 0.49 | 2104 | 16.04 | 0.51 | < 10^-4^ |
| Ag04450 | normal | 7359 | 6.97 | 0.21 | 4053 | 9.16 | 0.34 | < 10^-4^ | 617 | 46.96 | 1.61 | 547 | 49.01 | 1.63 | 0.1848 | 499 | 37.09 | 1.71 | 366 | 32.66 | 1.91 | 0.9513 | 1304 | 11.11 | 0.63 | 2251 | 20.43 | 0.63 | < 10^-4^ |
| Ag09309 | normal | 7339 | 9.29 | 0.24 | 4027 | 11.40 | 0.35 | < 10^-4^ | 623 | 52.57 | 1.56 | 596 | 54.31 | 1.51 | 0.2084 | 511 | 43.15 | 1.69 | 363 | 39.98 | 1.96 | 0.8907 | 1303 | 14.51 | 0.68 | 2311 | 23.78 | 0.60 | < 10^-4^ |
| Ag09319 | normal | 7117 | 7.27 | 0.22 | 3853 | 9.81 | 0.36 | < 10^-4^ | 553 | 48.76 | 1.74 | 490 | 51.05 | 1.79 | 0.1807 | 463 | 39.38 | 1.80 | 337 | 36.06 | 2.05 | 0.8828 | 1258 | 12.46 | 0.68 | 2091 | 23.24 | 0.69 | < 10^-4^ |
| Ag10803 | normal | 7604 | 7.77 | 0.23 | 4283 | 10.24 | 0.35 | < 10^-4^ | 693 | 51.19 | 1.49 | 640 | 53.17 | 1.46 | 0.1695 | 553 | 41.69 | 1.65 | 416 | 37.75 | 1.86 | 0.9441 | 1367 | 12.30 | 0.66 | 2502 | 23.30 | 0.62 | < 10^-4^ |
| Fibrobl | normal | 7156 | 9.05 | 0.24 | 3881 | 11.39 | 0.37 | < 10^-4^ | 639 | 54.80 | 1.53 | 579 | 55.89 | 1.53 | 0.3147 | 517 | 44.02 | 1.69 | 371 | 41.90 | 1.96 | 0.797 | 1267 | 14.43 | 0.70 | 2188 | 25.22 | 0.66 | < 10^-4^ |
| HAEpiC | normal | 7331 | 6.62 | 0.21 | 4026 | 8.78 | 0.34 | < 10^-4^ | 615 | 47.10 | 1.65 | 559 | 50.66 | 1.68 | 0.0676 | 510 | 36.50 | 1.72 | 362 | 34.85 | 2.02 | 0.7205 | 1298 | 10.05 | 0.62 | 2295 | 22.19 | 0.67 | < 10^-4^ |
| HCF | normal | 6773 | 5.98 | 0.21 | 3553 | 7.81 | 0.34 | < 10^-4^ | 522 | 47.48 | 1.80 | 452 | 48.00 | 1.85 | 0.4240 | 450 | 34.95 | 1.77 | 324 | 35.26 | 2.13 | 0.4675 | 1196 | 9.52 | 0.64 | 1945 | 20.60 | 0.71 | < 10^-4^ |
| HCM | normal | 7522 | 6.37 | 0.21 | 4183 | 8.37 | 0.33 | < 10^-4^ | 648 | 47.29 | 1.61 | 570 | 49.31 | 1.66 | 0.1872 | 529 | 36.96 | 1.69 | 389 | 33.20 | 1.92 | 0.9281 | 1348 | 9.84 | 0.63 | 2389 | 20.62 | 0.65 | < 10^-4^ |
| HEEpiC | normal | 7232 | 6.43 | 0.21 | 3950 | 8.39 | 0.33 | < 10^-4^ | 608 | 45.70 | 1.63 | 535 | 48.71 | 1.70 | 0.0922 | 488 | 34.05 | 1.69 | 352 | 33.65 | 1.96 | 0.562 | 1288 | 10.49 | 0.64 | 2175 | 20.43 | 0.66 | < 10^-4^ |
| HIPEpiC | normal | 7282 | 6.54 | 0.20 | 3999 | 9.13 | 0.34 | < 10^-4^ | 581 | 45.88 | 1.68 | 524 | 49.33 | 1.72 | 0.0755 | 484 | 35.32 | 1.73 | 356 | 33.55 | 1.95 | 0.7564 | 1296 | 10.26 | 0.61 | 2185 | 20.53 | 0.65 | < 10^-4^ |
| HMEC | normal | 7276 | 8.23 | 0.24 | 3984 | 10.70 | 0.38 | < 10^-4^ | 615 | 52.15 | 1.61 | 541 | 53.92 | 1.71 | 0.2315 | 503 | 41.27 | 1.75 | 364 | 39.97 | 2.07 | 0.6856 | 1296 | 13.84 | 0.71 | 2219 | 24.98 | 0.70 | < 10^-4^ |
| HNPCEpiC | normal | 7485 | 6.33 | 0.20 | 4141 | 8.07 | 0.32 | < 10^-4^ | 618 | 46.01 | 1.63 | 556 | 47.19 | 1.67 | 0.3054 | 505 | 34.63 | 1.71 | 373 | 31.55 | 1.91 | 0.8868 | 1337 | 9.32 | 0.59 | 2340 | 20.00 | 0.63 | < 10^-4^ |
| HRCEpiC | normal | 7009 | 5.59 | 0.21 | 3759 | 7.53 | 0.34 | < 10^-4^ | 549 | 45.54 | 1.74 | 477 | 46.45 | 1.83 | 0.3611 | 456 | 34.75 | 1.81 | 324 | 31.33 | 2.07 | 0.891 | 1243 | 8.68 | 0.60 | 2018 | 18.53 | 0.67 | < 10^-4^ |
| HSMMtube | normal | 7388 | 11.56 | 0.25 | 4119 | 15.29 | 0.40 | < 10^-4^ | 689 | 57.60 | 1.45 | 618 | 59.65 | 1.48 | 0.1625 | 540 | 49.25 | 1.61 | 398 | 45.43 | 1.88 | 0.9369 | 1319 | 18.12 | 0.73 | 2340 | 31.75 | 0.71 | < 10^-4^ |
| NHBE | normal | 7392 | 6.93 | 0.22 | 4059 | 9.04 | 0.34 | < 10^-4^ | 628 | 48.36 | 1.62 | 554 | 50.94 | 1.68 | 0.1309 | 513 | 37.23 | 1.70 | 377 | 37.07 | 1.96 | 0.5316 | 1312 | 11.29 | 0.66 | 2276 | 22.60 | 0.68 | < 10^-4^ |
| Skmc | normal | 7259 | 6.72 | 0.23 | 3958 | 8.74 | 0.35 | < 10^-4^ | 648 | 50.51 | 1.57 | 590 | 52.71 | 1.56 | 0.1601 | 525 | 39.89 | 1.67 | 390 | 37.92 | 1.93 | 0.784 | 1300 | 10.80 | 0.66 | 2273 | 22.81 | 0.68 | < 10^-4^ |
